# High phosphorus mediated the release of C‐X‐C motif chemokine ligand 8 in valvular interstitial cells‐induced endothelial‐to‐mesenchymal transition via miR‐214/phosphatase and tensin homolog to promote valvular calcification in chronic kidney disease

**DOI:** 10.1002/ctm2.733

**Published:** 2022-05-23

**Authors:** Li‐ting Wang, Yu‐jia Wang, Yu‐xia Zhang, Si‐jie Chen, Zi‐Xiao Liu, Jing‐yuan Cao, Wei‐jie Ni, Tao‐tao Tang, Ri‐ning Tang, Xiao‐liang Zhang, Bi‐cheng Liu

**Affiliations:** ^1^ Institute of Nephrology Zhong Da Hospital Southeast University School of Medicine Nanjing China; ^2^ State Key Laboratory for Modification of Chemical Fibers and Polymer Materials International Joint Laboratory for Advanced Fiber and Low‐dimension Materials College of Materials Science and Engineering Donghua University Shanghai China; ^3^ Institute of Nephrology NanJing LiShui People's Hospital Zhongda Hospital Lishui Branch Southeast University School of Medicine Nanjing China

Dear Editor,

We report here that high phosphorus (HP) caused the conversion of quiescent valvular interstitial cells (qVICs) to activated myofibroblast‐like VICs (aVICs) with CXCL8 releasing, and CXCL8‐induced valvular endothelial cells (VECs)’ endothelial‐to‐mesenchymal transition (EndMT) via miR‐214‐3p/phosphatase and tensin homolog (PTEN) pathway and thus contributed to valvular calcification (VC) in chronic kidney disease (CKD).

Accelerated and premature VC is a hallmark of patients with CKD.^1^ Extracellular matrix (ECM) accumulation and multi‐cells activation are the characteristics of VC in the early stage.^2^ Previous study suggested that a long‐term HP‐diet could aggravate ECM accumulation in valves of CKD rats.^3^ Furthermore, canine valvular interstitial cells (VICs) were given ‘starvation treatment’ (incubated without serum) for 12 h to transform into qVICs. qVICs were treated with phosphorus at different concentrations and for different lengths of time. VICs incubated with HP (3.6 mmol/L) exhibited an obvious increase in the mRNA levels of α‐smooth muscle actin (α‐SMA), smoothelin and vimentin at 24 h post‐treatment (Figure 1A,B). The increase in α‐SMA, smoothelin and vimentin reflected that qVICs have transformed into aVICs. Then, we used C57/Bj mice to establish a CKD model by feeding on a 6‐week 0.2% adenine diet followed by a 10‐week HP diet (P:1.8%, HP‐diet) or a 10‐week normal phosphorus diet (P:0.9%, NP‐diet). According to Alcian blue staining, HP‐diet increased valve ECM accumulation compared with NP‐diet (Figure 1C,D). An increased number of VICs expressing vimentin on the valves could be observed, implying that HP‐diet could accelerate the conversion of qVICs to aVICs (Figure 1E,F). However, HP could not further promote the conversion of aVICs to osteoblast VICs (oVICs) (Figure S1A,B and Figure 1G,H).

Then, a co‐culture system was established to investigate the cell crosstalk between VICs and VECs. In the transwell cabin, VICs (4×10^4) were cultured in the upper chamber with a membrane pore size of 0.4 μm for 24 h with HP (Figure S2A). Next, VICs in the upper chamber were co‐cultured with human umbilical vein endothelial cells (HUVECs)/VECs in the lower chamber. HUVECs/VECs in the lower chamber were collected after 48 h of co‐cultivation. HUVECs co‐cultured with aVICs were found to undergo EndMT compared with HUVECs co‐cultured with qVICs, characterised with downregulated endothelial markers (vascular endothelial [VE]‐cadherin, CD31, von Willebrand factor [vWF]) and upregulated mesenchymal/mesenchymal stem cell markers (α‐SMA, fibroblast specific protein 1 [FSP1], N‐cadherin, CD10, CD44) in the protein levels (Figure S2B,C). HUVECs co‐cultured with qVICs maintained endothelial phenotype. Besides, a transwell assay was established for co‐cultivation of canine VICs and canine VECs (Figure S3A‐C). A similar phenomenon occurred in canine VECs (Figure S3D). These revealed that canine VECs underwent EndMT in a transwell assay. In summary, aVICs activated by HP could induce EndMT. However, whether HP could cause VECs to undergo EndMT needs to be considered. Canine VECs were stimulated using HP (Figure S4A). The mRNA levels of endothelial markers and mesenchymal markers did not exhibit significant changes (Figure S4B,C), reflecting that HP cannot directly cause VECs’ EndMT in vitro.

Previously, it has been reported that the pro‐inflammatory factors in calcified valves were elevated in non‐CKD patients with calcified aortic valve disease, such as transforming growth factor beta 1 (TGF‐β1), CXCL6, CXCL8, tumor necrosis factor‐alpha (TNF‐α) and interleukin‐1β (IL‐1β).^4^, ^5^, ^6^ We speculated that these pro‐inflammatory factors might be the cause of EndMT. Compared with qVICs, the mRNA levels of TGF‐β1, CXCL6, CXCL8 and TNF‐α in aVICs were increased (Figure 2A). In the transwell assay, infliximab, tocilizumab, Disitertide (P144) and Reparixin were employed to inhibit the TNF‐α, CXCL6, TGF‐β1 and CXCL8 receptors of HUVECs, respectively. Disitertide (P144) and Reparixin could partly relieve EndMT (Figure 2B‐E). These results suggested that CXCL8 and/or TGF‐β1 participated in aVICs‐induced EndMT. The levels of canine CXCL8 and canine TGF‐β1 in the lower chamber of the transwell assay were also tested (Figure S5A‐C). The concentration of CXCL8 was found to reach 2–4 ng/ml, which was much higher than the concentration of canine TGF‐β1 (∼300 pg/ml). Meanwhile, canine VECs were treated by TGF‐β1 (300 pg/ml), CXCL8 (4 ng/ml) and CXCL8+TGF‐β1 (Figure S5D). The results revealed that canine VECs treated by CXCL8 and CXCL8+TGF‐β1 underwent EndMT. However, there were no significant changes in HUVECs treated by TGF‐β1. Thus, CXCL8 could be a crucial regulator between the crosstalk of VICs and VECs. In the mice model of CKD, compared with the NP‐diet group, valvular CXCL2, the mouse CXCL8 functional homolog^7^ was increased in the HP‐diet group (Figure S6A,B). This suggested that HP‐diet could promote the release of CXCL2 from the valves. Additionally, Tek‐EGFP‐PolyA mice were established with an enhanced green fluorescent protein (EGFP), which was controlled by the endothelial‐specific TEK promoter (Figure S7A,B). Tek‐EGFP‐PolyA mice were used to establish the CKD model. The mice in the AAV9‐sm22a‐CXCL2 group were given a tail‐vein injection of AAV9‐sm22a‐CXCL2 to inhibit the release of CXCL2 by VICs after a 6‐week adenine diet. The mice of the Reparixin group were given an abdominal injection of Reparixin to inhibit CXCL2 function by binding CXCR1/CXCR2 (CXCL8 receptor) noncompetitively (Figure S6C). In the group of AAV9‐sm22a‐CXCL2 and Reparixin, the numbers of VECs‐EGFP co‐localized with α‐SMA and FSP1 decreased compared with CKD group (Figure S6D,E). In summary, aVICs induced EndMT by releasing CXCL8 in CKD with VC.

**FIGURE 1:**
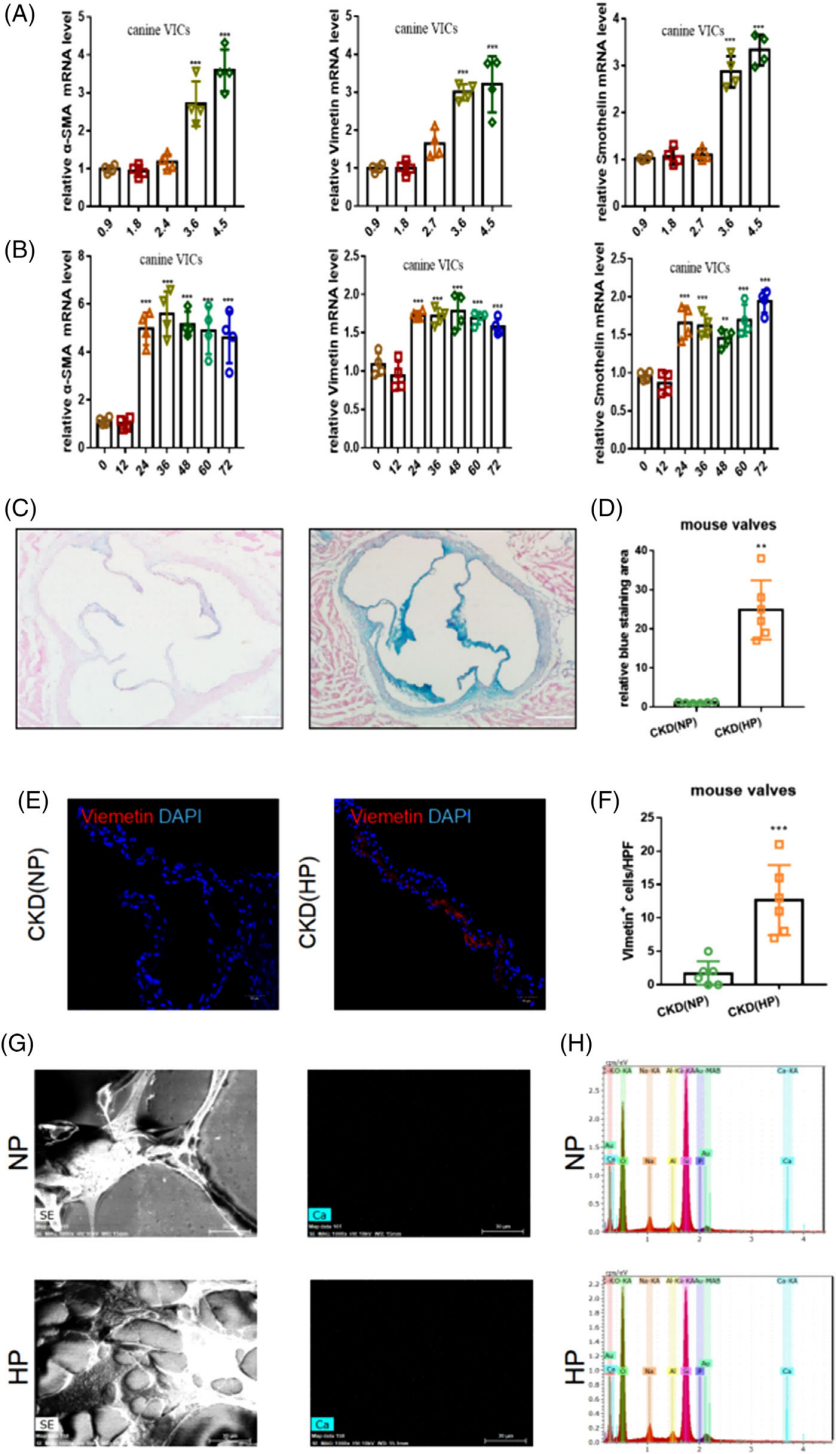
High phosphorous (HP) induced valvular interstitial cell (VIC) activation. (A) HP (0.9, 1.8, 2.7, 3.6 4.5 mmol/L) was intervened in VICs for 24 h. Each group was illustrated as means±SE and repeated four times. ^***^, *p *< .001 versus CTL. (B) HP (3.6 mmol/L) was intervened in VIC for 0, 12, 24, 36, 48, 60 and 72 h. Each group was presented as means± SE and repeated four times. ^**^, *p *< .01 versus CTL; ^***^, *p *< .001 versus CTL. (C‐F) C57/Bj mice were used to establish the chronic kidney disease (CKD) model through 6 week‐adenine diet (0.2%) with/without 10 week‐HP diet (1.5%). Two groups were CKD (NP) and CKD (HP). (C,D) The Alcian blue staining of arotic valves in CKD mice was counted. Each group was exhibited as means±SE and repeated six times. (E,F) Viemetin+ cell (Red) was recorded. Each group was exhibited as means±SE and repeated six times. ^***^, *p *< .001 versus CKD (NP). (G,H) SEM was used to observe VICs, and elemental analysis was performed to observe whether VICs synthesised calcium particles. CTL, the control; SEM, scanning electron microscope

**FIGURE 2:**
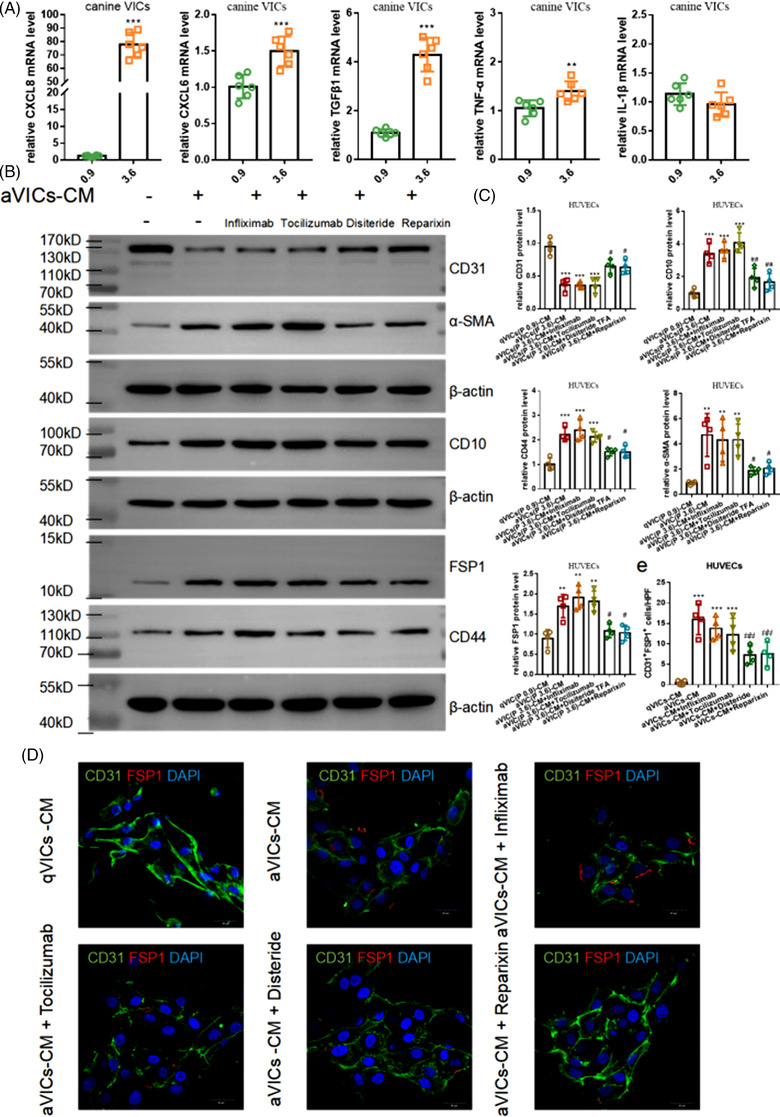
CXCL8 and transforming growth factor beta 1 (TGFβ1) released by activated myofibroblast‐like valvular interstitial cells (aVICs) may be the factor inducing endothelial cells (ECs) endothelial‐to‐mesenchymal transition (EndMT). The following experiment was conducted to validate which aVIC released mediates ECs EndMT. (A) The mRNA levels of CXCL8, CXCL6, tumor necrosis factor‐alpha (TNF‐α), interleukin 1‐β (IL1‐β) and transforming growth factor β1 (TGF‐β1) in aVICs and quiescent valvular interstitial cells (qVICs) were examined. The phosphorus concentrations of the intervention were .9 and 3.6 mmol/L. Each group was presented as means±SE and repeated six times. ^**^, *p *< .01 versus .9; ^***^, *p *< .001 versus .9. (B) In the transwell assay, infliximab, tocilizumab, Disitertide (P144) and Reparixin were used to inhibit the TNF‐α, CXCL6, TGF‐β‐1 and CXCL8 receptors of human umbilical vein endothelial cells (HUVECs), respectively. Endothelial markers and interstitial markers were examined in HUVECs. Each group was exhibited as means±SE and repeated four times. ^**^, *p *< .01 versus qVICs (P .9)‐CM; ^***^, *p *< .001 versus qVICs (P .9)‐CM; ^#^, *p* < 0.05 versus aVICs (P 3.6)‐CM; ^##^, *p* < .05 versus aVICs (P 3.6)‐CM. (D,E) The immunofluorescence of CD31 and fibroblast specific protein 1 (FSP1). HUVECs co‐expressing CD31 and FSP1 were counted. Each group was exhibited as means±SE and repeated four times. ^***^, *p *< .001 versus qVICs (P .9)‐CM; ^##^, *p* < .05 versus aVICs (P 3.6)‐CM

Then, we studied how CXCL8 released by aVICs induced VECs EndMT. Specifically, the expression profiles of miRNAs in HUVEC co‐cultured with aVIC and qVIC were subsequently determined (Figure 3A). The results confirmed that several HUVECs miRNAs are regulated by aVICs characteristic with 1897 upregulated miRNAs and 2308 downregulated miRNAs (Figure 3B). Upon CXCL8 stimulation, compared with the control (CTL) group, the levels of miR‐214‐3p increased and this increase could be attenuated by Reparixin (Figure 3C). This is consistent with the results of the miRNAs sequencing in the co‐culture system. Meanwhile, miR‐214‐3p‐inhibitor could inhibit CXCL8‐mediated EndMT (Figure 3D‐G). Dual‐luciferase reporter assay also verified that PTEN was the direct target of miR‐214‐3p (Figure S8A,B). miR‐214‐3p‐inhibitor could restore PTEN expression (Figure S8C,D). PTEN was reported to negatively regulate intracellular protein levels of AKT/PKB signalling pathways.^8^ Besides, CXCL8 could induce the activation of the AKT pathway. EndMT is driven by transcription factors such as SNAI1 and ZEB1 and is a cell reprogramming process regulated by multiple molecular pathways, especially PI3K/AKT/MTOR (mammalian target of rapamycin).^9^ OE‐PTEN could partially restore CD31 expression and repress the expression of mesenchymal markers (CD10, CD44, FSP1 and α‐SMA) (Figure 3D,E). Moreover, the number of HUVECs co‐expressing CD31 and FSP1 decreased when treated with OE‐PTEN or miR‐214‐3p‐inhibitor (Figure 3F,G). Furthermore, endothelial cells’ (ECs) miR‐214‐3p expression was inhibited with AAV9‐TIE‐miR‐214 to further demonstrate the role of miR‐214‐3p in vivo. After 6 weeks of adenine diet, Tek‐EGFP‐PolyA mice were injected with AAV9‐TIE‐miR‐214‐3p through the tail vein (Figure S9A). In the mice model of CKD, the number of VECs co‐expressing miR‐214‐3p increased compared with CTL, and this increase could be inhibited by TIE2‐AAV‐miR‐214 (Figure S9B,C). The numbers of VECs‐EGFP co‐localized with FSP1 decreased compared with the CKD group, suggesting that the inhibition of miR‐214‐3p could alleviate EndMT (Figure S9D,E). To conclude, CXCL8 induced EndMT through miR‐214‐3p/PTEN/Akt pathway in CKD with VC.

**FIGURE 3:**
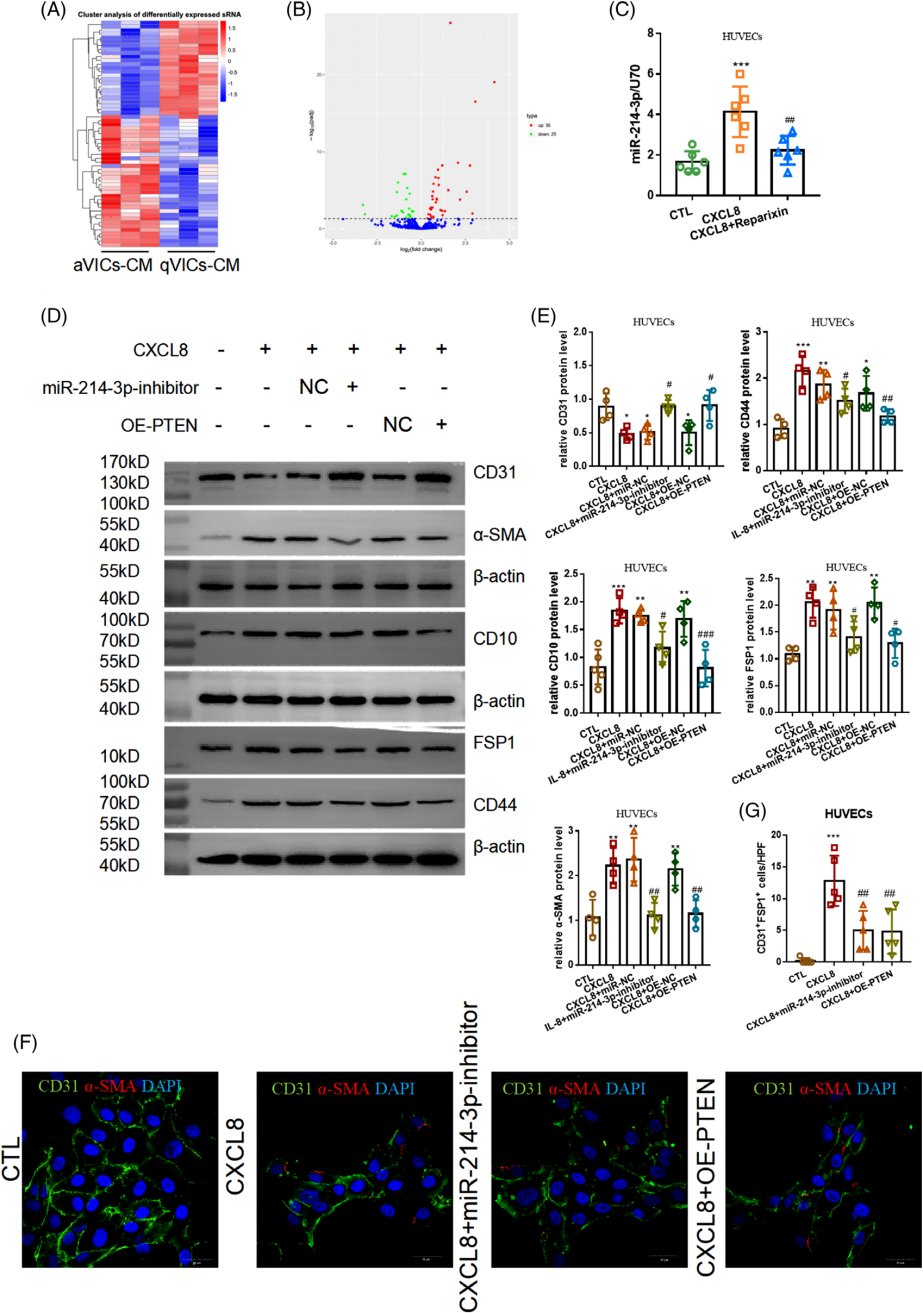
CXCL8 released by activated myofibroblast‐like valvular interstitial cells (aVICs) induced endothelial‐to‐mesenchymal transition (EndMT) of endothelial cells (ECs) by upregulating miR‐214‐3p. (A) miRNA sequencing in human umbilical vein endothelial cells (HUVECs) by aVICs‐CM versus quiescent valvular interstitial cells (qVICs)‐CM. *n *= 3, each group. (B) Volcano map. (C) The level of miR‐214‐3p in HUVECs was detected upon CXCL8 (4 ng/ml) with/without Reparixin. Each group was presented as means±SE and repeated six times. ^***^, *p *< .001 versus CTL; ^##^, *p *< .01 versus CXCL8. (D,E) The endothelial markers and interstitial markers of HUVECs were detected upon CXCL8 (4 ng/ml) with/without miR‐214‐3p‐inhibitor and OE‐PTEN. Each group was exhibited as means±SE and repeated six times. ^*^, *p *< .05 versus CTL; ^**^, *p *< .01 versus CTL; ^***^, *p *< .001 versus CTL; ^#^, *p *< .05 versus CXCL8; ^##^, *p *< .01 versus CXCL8. (F,G) The HUVECs co‐expressing CD31 and fibroblast specific protein 1 (FSP1) were counted. Each group was illustrated as means±SE and repeated four times. ^**^, *p *< .01 versus CTL; ^***^, *p *< .001 versus CTL; ^##^, *p *< .01 versus CXCL8. CTL, the control

**FIGURE 4:**
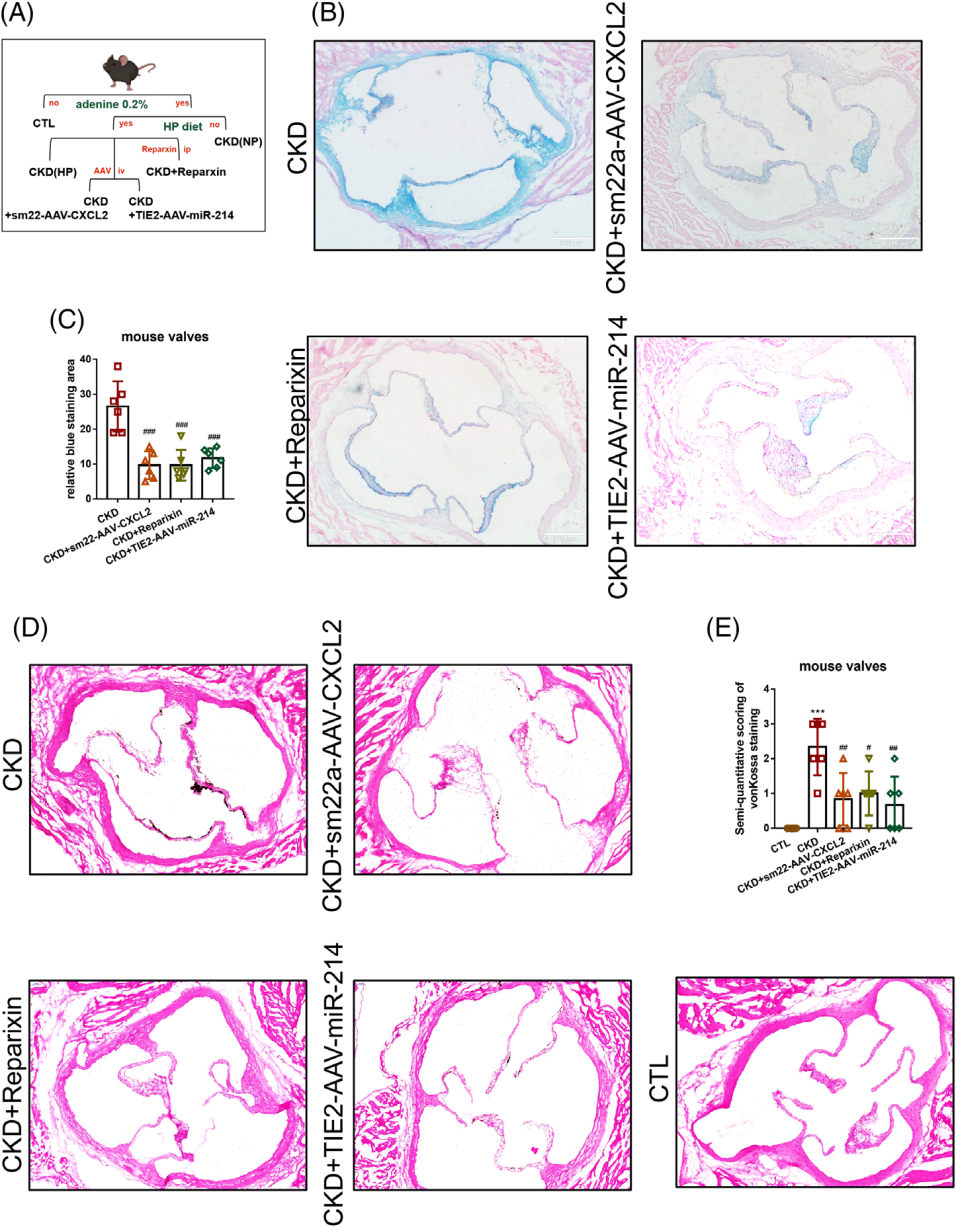
Inhibition of the CXCL8/miR‐214 pathway could attenuate valvular extracellular matrix (ECM) accumulation and VC. (A) The diagram of TEK^+^ mouse. (B,C) This was Alcian blue staining and the area of blue staining was counted. Each group was exhibited as means±SE and repeated six times. ^***^, *p *< .001 versus chronic kidney disease (CKD). (D,E). This was Vonkossa staining and the area of black staining was counted. Each group was illustrated as means±SE and repeated six times. ^*^, *p *< .05 versus CKD; ^**^, *p *< .01 versus CKD. VC, valvular calcification

ECs contribute to myofibroblast activation by undergoing EndMT and have been identified to participate in ECM accumulation and valvular calcification.^10^ We further investigated whether blocking the CXCL8/miR‐214‐3p/PTEN/Akt pathway could alleviate ECM accumulation. Therefore, three intervention groups with TEK‐EGFP‐PolyA mice were designed: AAV9‐sm22a‐CXCL2 (inhibiting the expression of valve CXCL8 functional homolog CXCL2), Reparixin (non‐competitively antagonising CXCR1/CXCR2) and AAV9‐miR‐214‐3p (repressing endothelial miR‐214‐3p expression). The blue‐stained area of the valve decreased compared with the CKD+HP group based on Alcian blue staining (Figure 4A,B). Meanwhile, Vonkossa staining suggested that the calcification area of these three groups decreased compared with the CKD+HP group (Figure 4C,D). Therefore, inhibiting EndMT by blocking CXCL8/miR‐214‐3p could alleviate ECM accumulation and VC.

In conclusion, this study demonstrated that HP could cause the conversion of qVICs to aVICs and the release of CXCL8. The released CXCL8 further induced VECs EndMT via miR‐214‐3p/PTEN pathway. Our study provides a new insight on the formation of valvular calcification in CKD.

## Supporting information

SUPPORTING INFORMATION

SUPPORTING INFORMATION

SUPPORTING INFORMATION

SUPPORTING INFORMATION

SUPPORTING INFORMATION

SUPPORTING INFORMATION

SUPPORTING INFORMATION

SUPPORTING INFORMATION

SUPPORTING INFORMATION

SUPPORTING INFORMATION

SUPPORTING INFORMATION

SUPPORTING INFORMATION

SUPPORTING INFORMATION
