# Supplementary material for: High phosphorus mediated the release of C‐X‐C motif chemokine ligand 8 in valvular interstitial cells‐induced endothelial‐to‐mesenchymal transition via miR‐214/phosphatase and tensin homolog to promote valvular calcification in chronic kidney disease
Source: Clin Transl Med. 2022 May 23;12(5):e733. doi: 10.1002/ctm2.733 (PMC9126498; doi:10.1002/ctm2.733)
Supplement: Supplementary file 1 — SUPPORTING INFORMATION [file CTM2-12-e733-s009.pdf]

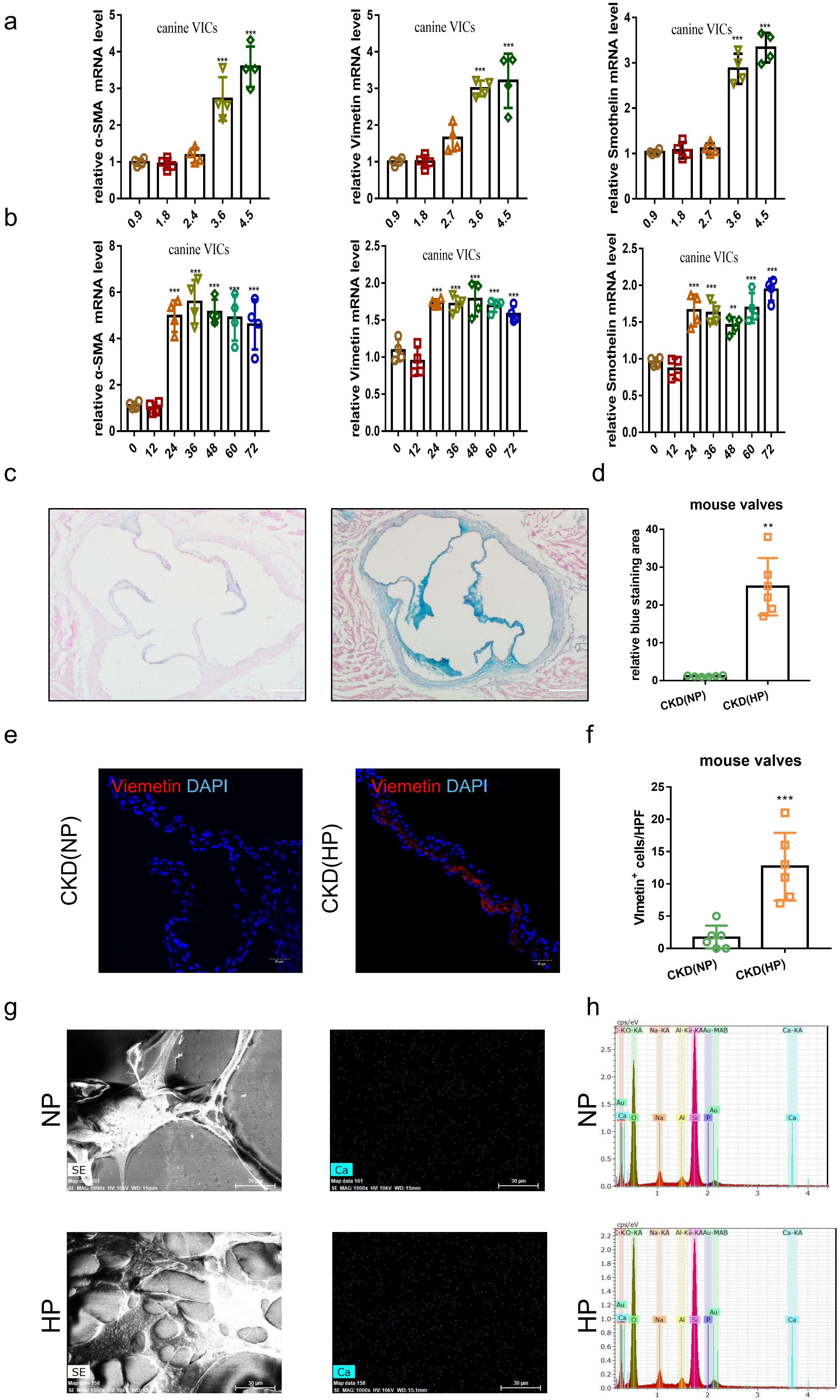

a
canine VICs
relative α-SMA mRNA level
relative Vimetin mRNA level
relative Smothelin mRNA level
0.9
1.8
2.4
2.7
3.6
4.5
***
b
canine VICs
0
12
24
36
48
60
72
**
c
d
mouse valves
relative blue staining area
CKD(NP)
CKD(HP)
e
Viemetin DAPI
f
Vimetin+ cells/HPF
g
NP
HP
SE
Ca
h
cps/eV
O-KA
Na-KA
Ca-KA
Au
O
Na
Al
Si
P

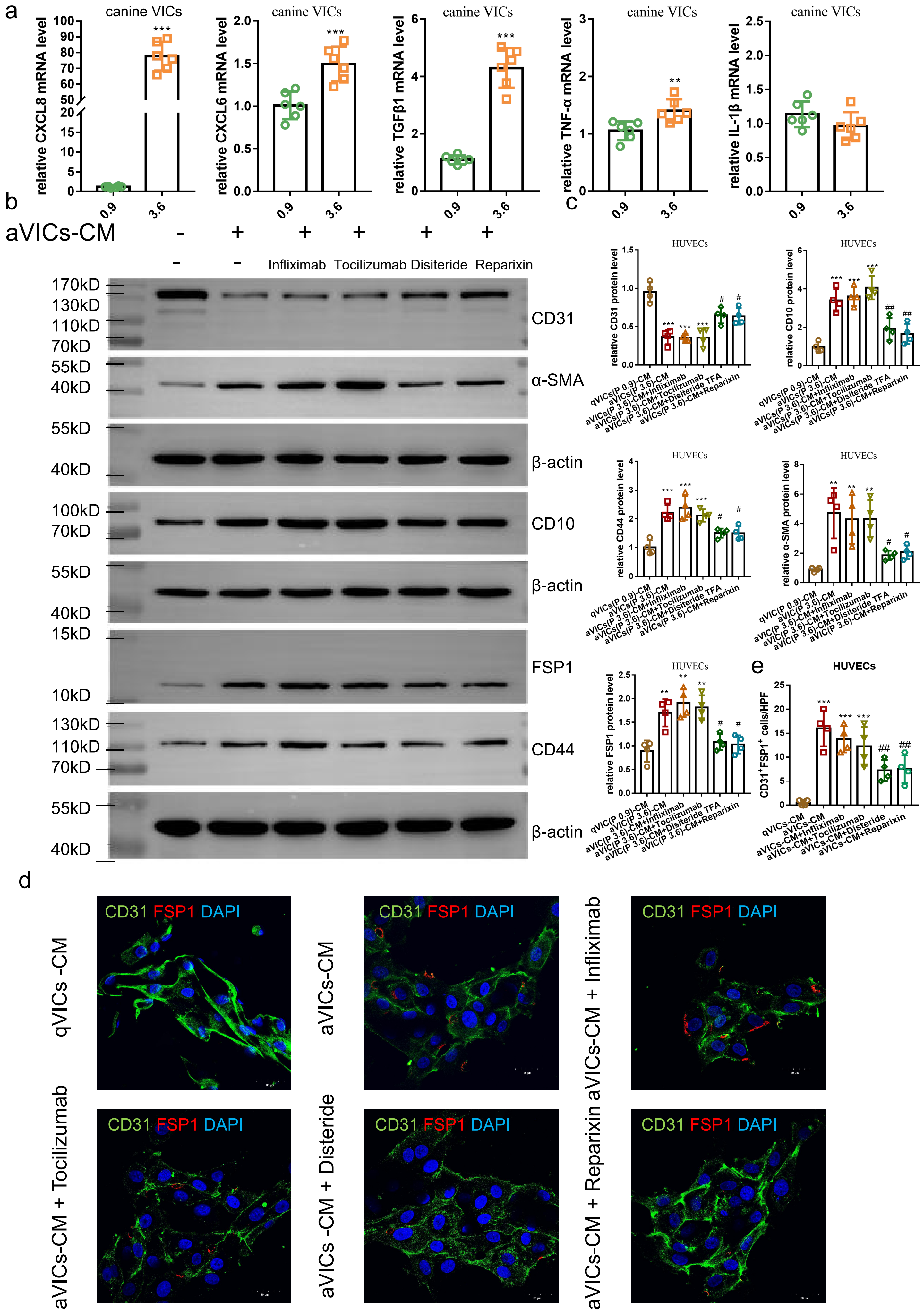
a
canine VICs
relative CXCL8 mRNA level
relative CXCL6 mRNA level
relative TGFβ1 mRNA level
relative TNF-α mRNA level
relative IL-1β mRNA level
0.9
3.6
b
aVICs-CM
Infliximab
Tocilizumab
Disiteride
Reparixin
170kD
130kD
110kD
70kD
55kD
40kD
100kD
15kD
10kD
CD31
α-SMA
β-actin
CD10
FSP1
CD44
c
HUVECs
relative CD31 protein level
relative CD10 protein level
relative CD44 protein level
relative α-SMA protein level
relative FSP1 protein level
qVICs(P 0.9)-CM
aVICs(P 3.6)-CM
aVICs(P 3.6)-CM+Infliximab
aVICs(P 3.6)-CM+Tocilizumab
aVICs(P 3.6)-CM+Disiteride TFA
aVICs(P 3.6)-CM+Reparixin
e
CD31+FSP1+ cells/HPF
qVICs-CM
aVICs-CM
aVICs-CM+Infliximab
aVICs-CM+Tocilizumab
aVICs-CM+Disteride
aVICs-CM+Reparixin
d
CD31 FSP1 DAPI
qVICs -CM
aVICs-CM
aVICs-CM + Infliximab
aVICs-CM + Tocilizumab
aVICs -CM + Disteride
aVICs-CM + Reparixin

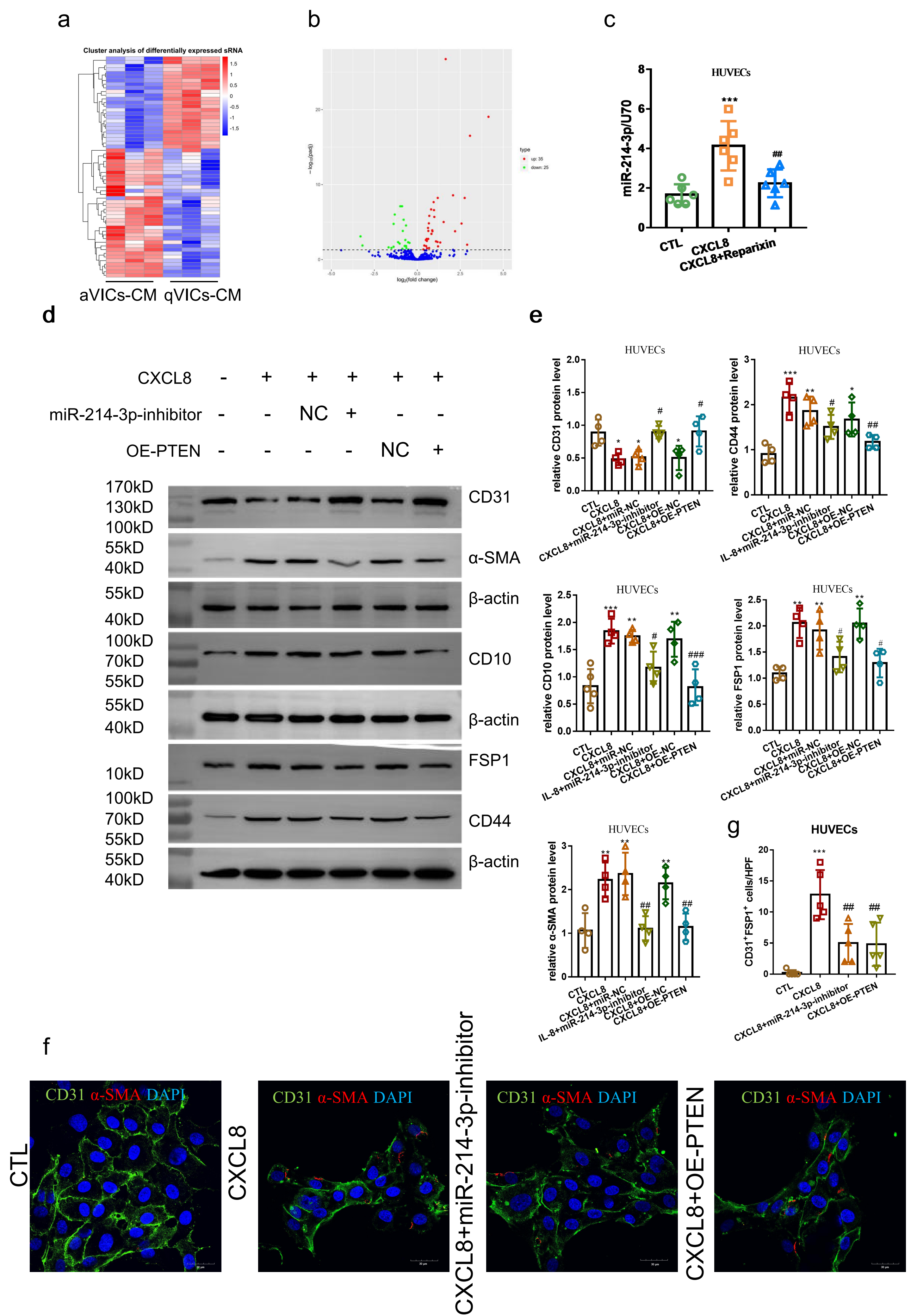

a
Cluster analysis of differentially expressed sRNA
aVICs-CM
qVICs-CM
b
type
up: 35
down: 25
log2(fold change)
c
HUVECs
miR-214-3p/U70
CTL
CXCL8
CXCL8+Reparixin
d
CXCL8
miR-214-3p-inhibitor
OE-PTEN
NC
CD31
α-SMA
β-actin
CD10
FSP1
CD44
e
HUVECs
relative CD31 protein level
relative CD44 protein level
relative CD10 protein level
relative FSP1 protein level
relative α-SMA protein level
CXCL8+miR-NC
CXCL8+miR-214-3p-inhibitor
CXCL8+OE-NC
CXCL8+OE-PTEN
IL-8+miR-214-3p-inhibitor
g
CD31+FSP1+ cells/HPF
f
CD31 α-SMA DAPI
CTL
CXCL8
CXCL8+miR-214-3p-inhibitor
CXCL8+OE-PTEN

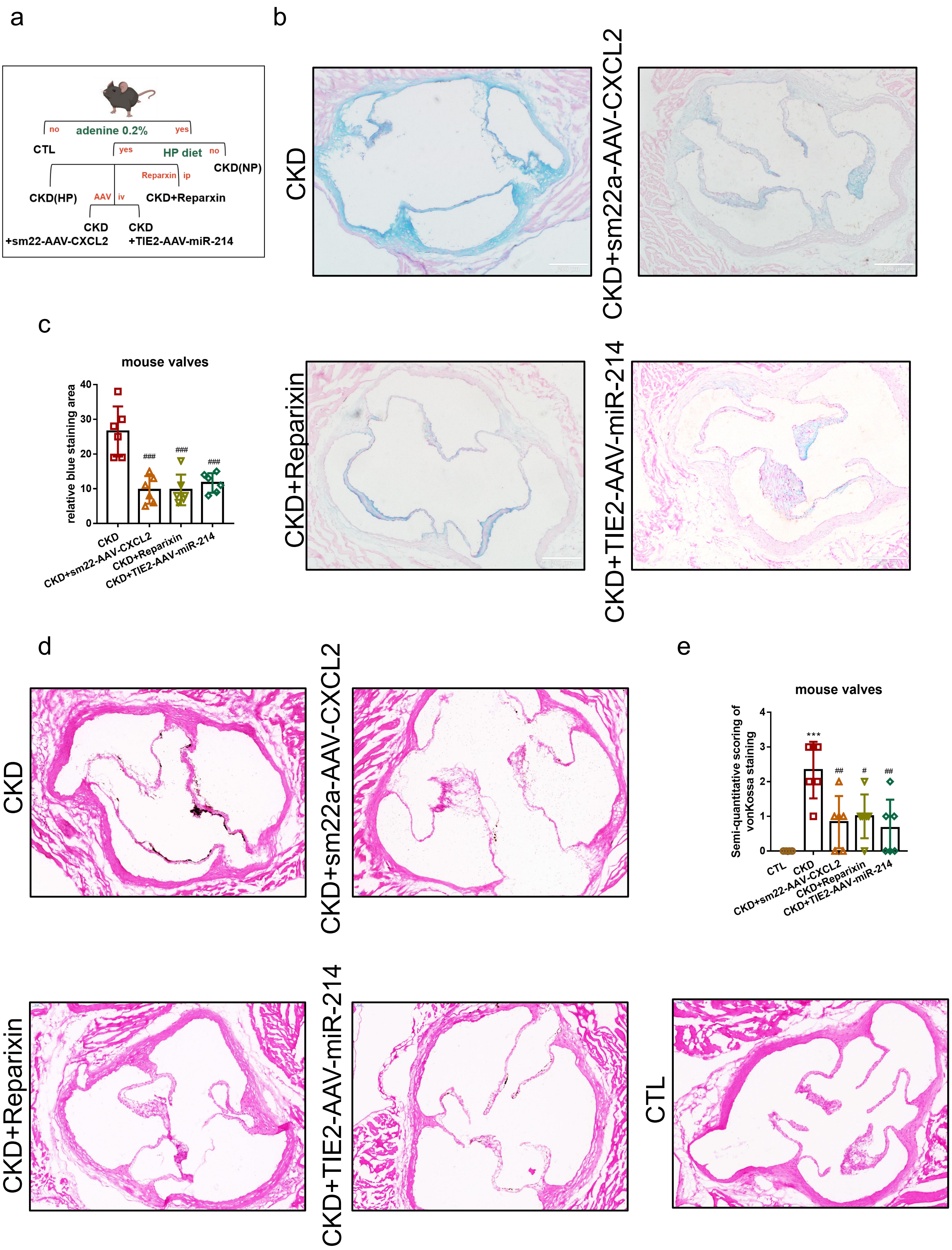
a
b
no
adenine 0.2%
yes
CTL
yes
HP diet
no
CKD(NP)
Reparxin
ip
CKD(HP)
AAV
iv
CKD+Reparxin
CKD
+sm22-AAV-CXCL2
CKD
+TIE2-AAV-miR-214
CKD
CKD+sm22a-AAV-CXCL2
c
mouse valves
relative blue staining area
40
30
20
10
0
###
###
###
CKD
CKD+sm22-AAV-CXCL2
CKD+Reparixin
CKD+TIE2-AAV-miR-214
CKD+Reparixin
CKD+TIE2-AAV-miR-214
d
CKD
CKD+sm22a-AAV-CXCL2
e
mouse valves
Semi-quantitative scoring of vonKossa staining
4
3
2
1
0
***
##
#
##
CTL
CKD
CKD+sm22-AAV-CXCL2
CKD+Reparixin
CKD+TIE2-AAV-miR-214
CKD+Reparixin
CKD+TIE2-AAV-miR-214
CTL

a

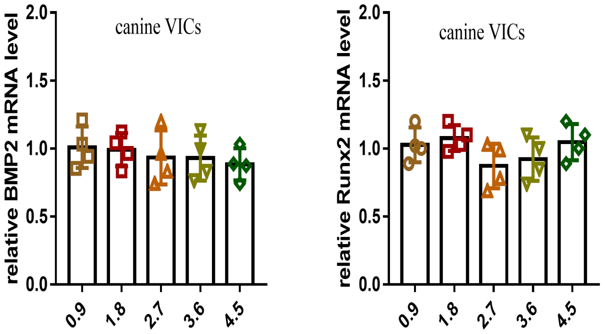
canine VICs
relative BMP2 mRNA level
2.0
1.5
1.0
0.5
0.0
0.9
1.8
2.7
3.6
4.5
canine VICs
relative Runx2 mRNA level
2.0
1.5
1.0
0.5
0.0
0.9
1.8
2.7
3.6
4.5

b

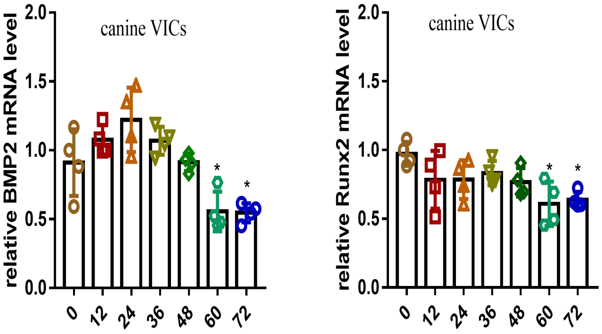
canine VICs
relative BMP2 mRNA level
2.0
1.5
1.0
0.5
0.0
0
12
24
36
48
60
72
*
*
canine VICs
relative Runx2 mRNA level
2.0
1.5
1.0
0.5
0.0
0
12
24
36
48
60
72
*
*

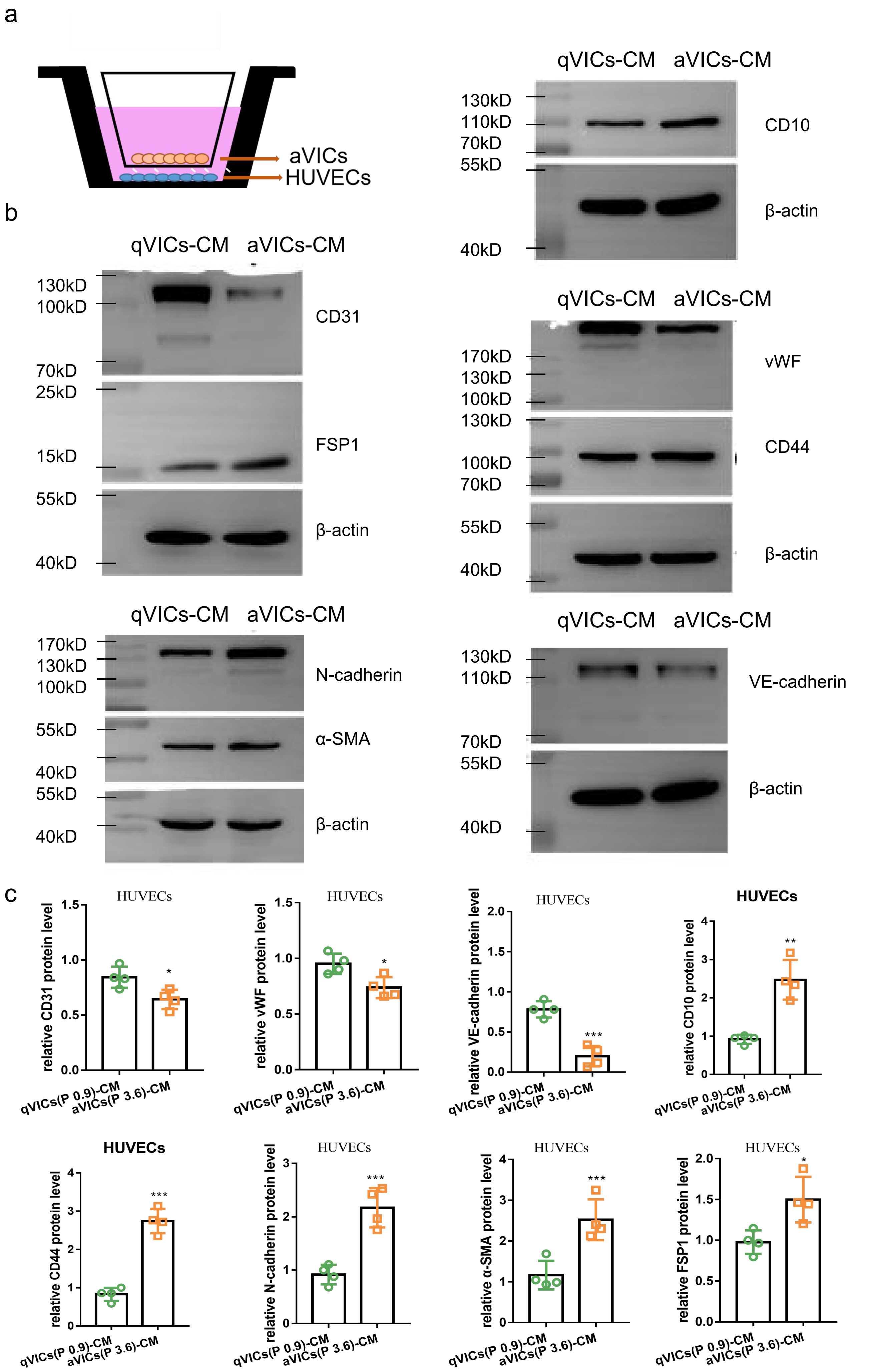

a
aVICs
HUVECs
b
qVICs-CM aVICs-CM
CD31
FSP1
β-actin
N-cadherin
α-SMA
CD10
vWF
CD44
VE-cadherin
130kD
110kD
100kD
70kD
55kD
40kD
25kD
15kD
170kD
c
HUVECs
relative CD31 protein level
relative vWF protein level
relative VE-cadherin protein level
relative CD10 protein level
relative CD44 protein level
relative N-cadherin protein level
relative α-SMA protein level
relative FSP1 protein level
qVICs(P 0.9)-CM
aVICs(P 3.6)-CM

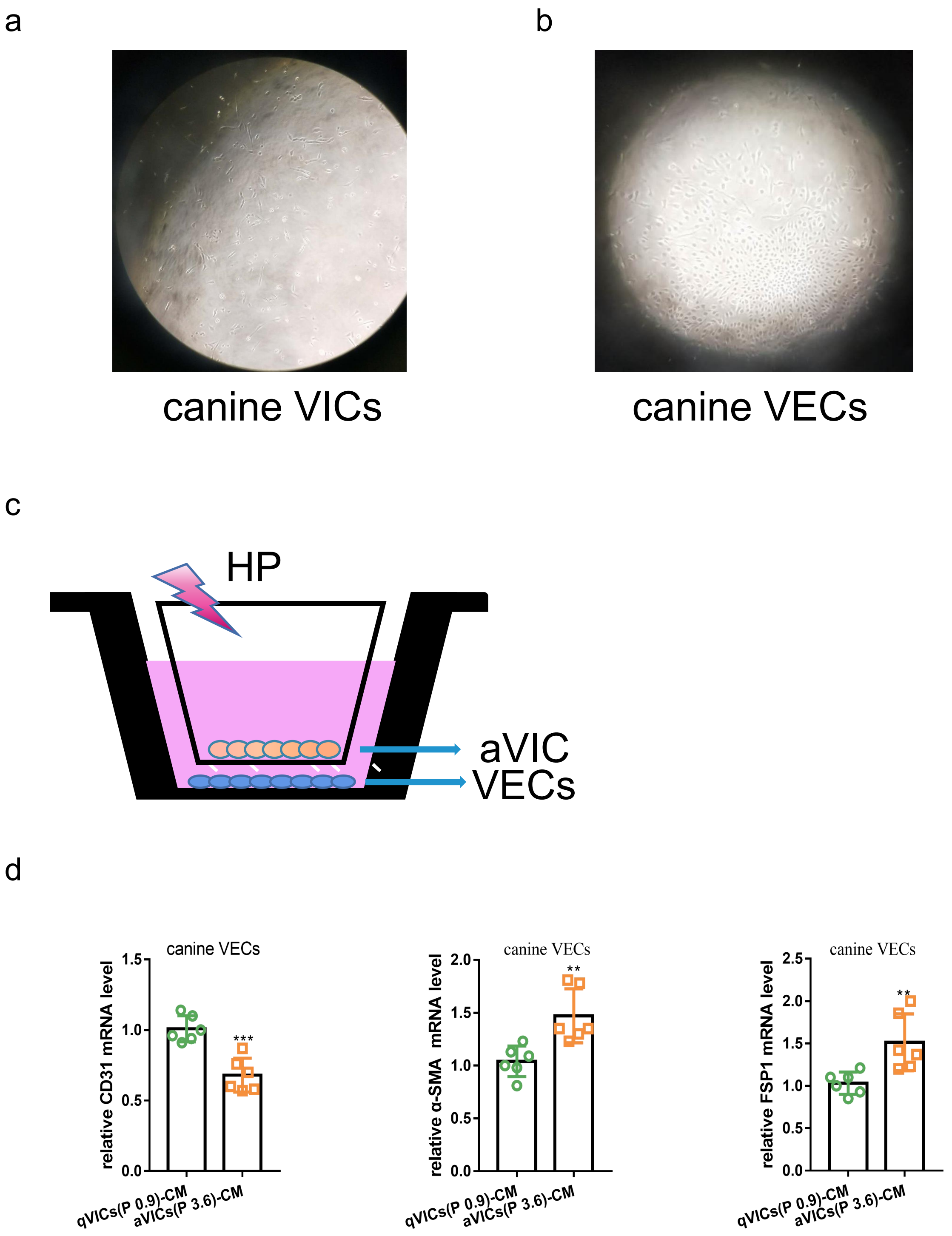
a
b
canine VICs
canine VECs
c
HP
aVIC
VECs
d
canine VECs
relative CD31 mRNA level
1.5
1.0
0.5
0.0
***
qVICs(P 0.9)-CM
aVICs(P 3.6)-CM
canine VECs
relative α-SMA mRNA level
2.0
1.5
1.0
0.5
0.0
**
qVICs(P 0.9)-CM
aVICs(P 3.6)-CM
canine VECs
relative FSP1 mRNA level
2.5
2.0
1.5
1.0
0.5
0.0
**
qVICs(P 0.9)-CM
aVICs(P 3.6)-CM

a

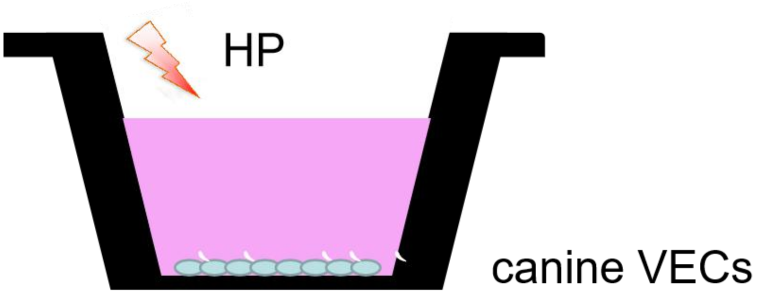
HP
canine VECs

b

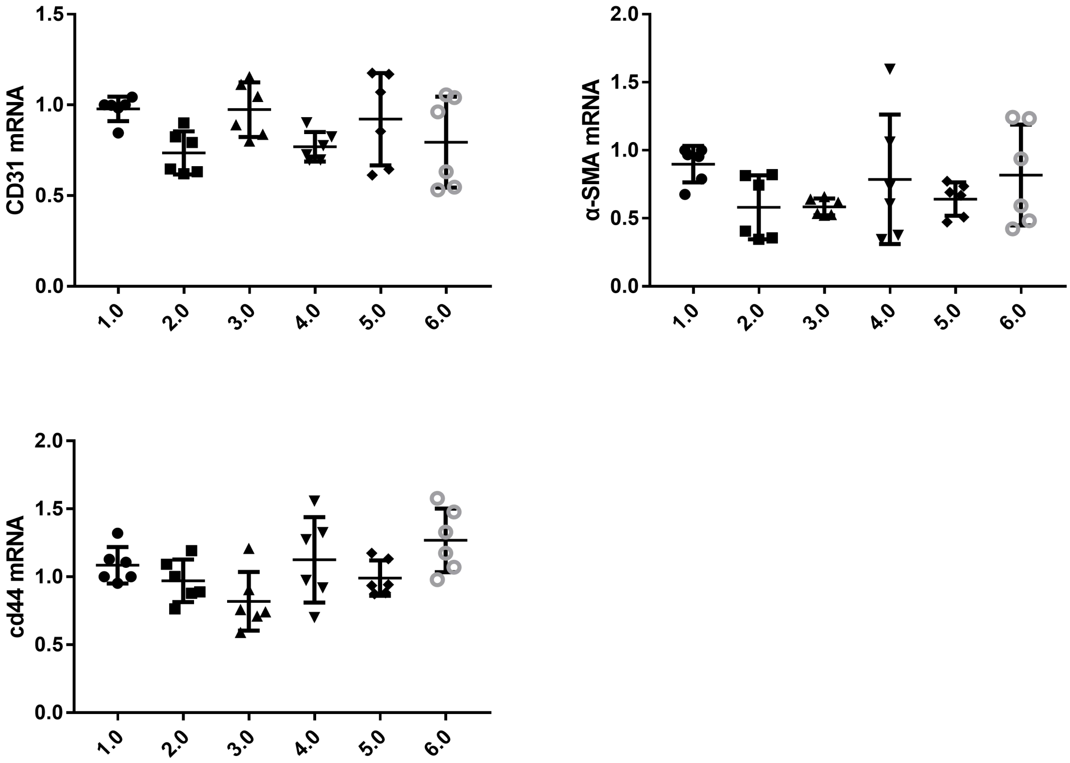
CD31 mRNA
1.5
1.0
0.5
0.0
1.0
2.0
3.0
4.0
5.0
6.0
α-SMA mRNA
2.0
1.5
1.0
0.5
0.0
1.0
2.0
3.0
4.0
5.0
6.0
cd44 mRNA
2.0
1.5
1.0
0.5
0.0
1.0
2.0
3.0
4.0
5.0
6.0

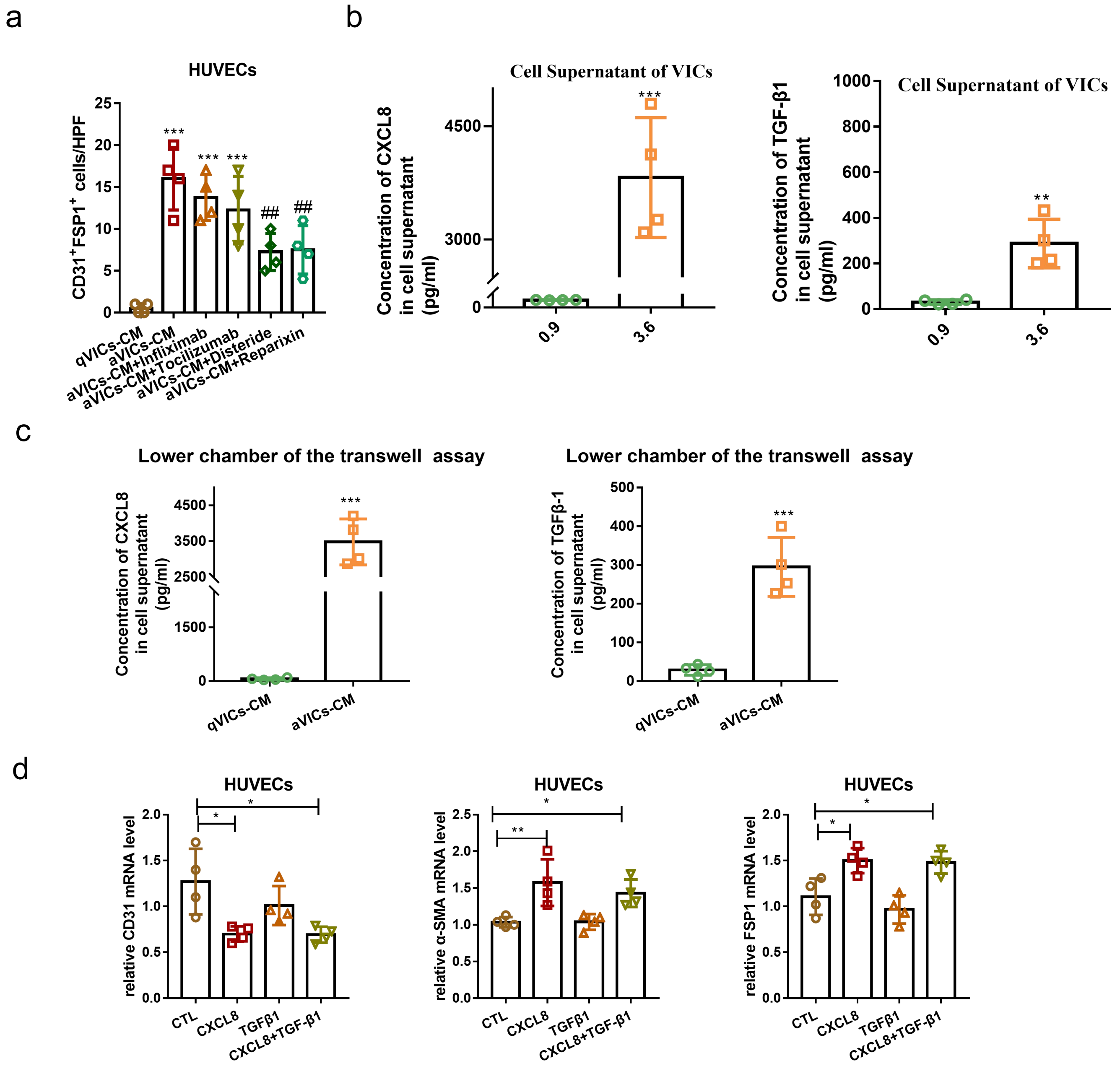

a
HUVECs
CD31+FSP1+ cells/HPF
qVICs-CM
aVICs-CM
aVICs-CM+Infliximab
aVICs-CM+Tocilizumab
aVICs-CM+Disteride
aVICs-CM+Reparixin
b
Cell Supernatant of VICs
Concentration of CXCL8 in cell supernatant (pg/ml)
Concentration of TGF-β1 in cell supernatant (pg/ml)
0.9
3.6
c
Lower chamber of the transwell assay
Concentration of CXCL8 in cell supernatant (pg/ml)
Concentration of TGFβ-1 in cell supernatant (pg/ml)
qVICs-CM
aVICs-CM
d
HUVECs
relative CD31 mRNA level
relative α-SMA mRNA level
relative FSP1 mRNA level
CTL
CXCL8
TGFβ1
CXCL8+TGF-β1

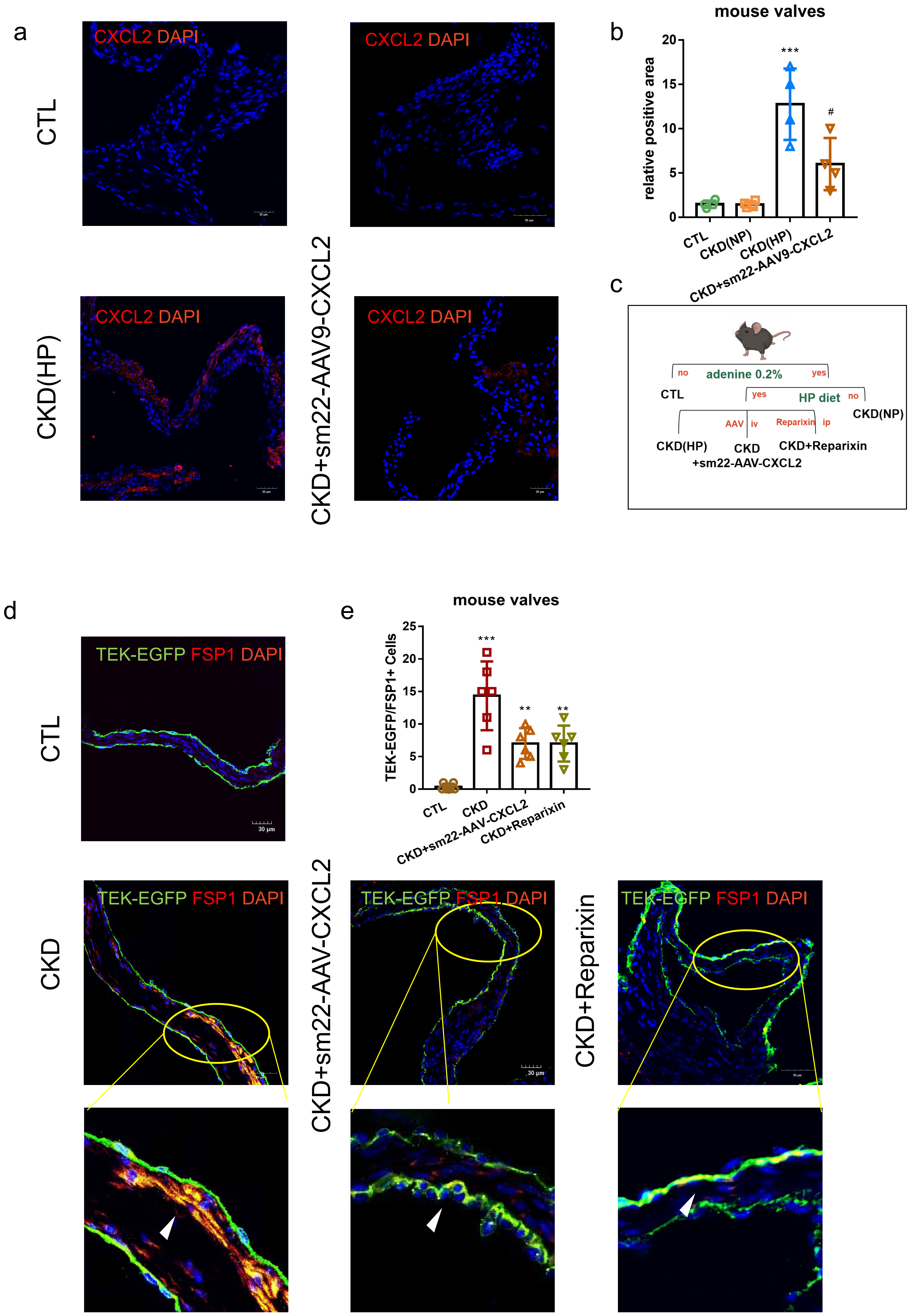

a
CTL
CXCL2 DAPI
CXCL2 DAPI
CKD(HP)
CXCL2 DAPI
CKD+sm22-AAV9-CXCL2
CXCL2 DAPI
b
mouse valves
relative positive area
***
#
CTL
CKD(NP)
CKD(HP)
CKD+sm22-AAV9-CXCL2
c
no
adenine 0.2%
yes
CTL
yes
HP diet
no
CKD(NP)
AAV
iv
Reparixin
ip
CKD(HP)
CKD
+sm22-AAV-CXCL2
CKD+Reparixin
d
CTL
TEK-EGFP FSP1 DAPI
30 μm
e
mouse valves
TEK-EGFP/FSP1+ Cells
***
**
**
CTL
CKD
CKD+sm22-AAV-CXCL2
CKD+Reparixin
CKD
TEK-EGFP FSP1 DAPI
CKD+sm22-AAV-CXCL2
TEK-EGFP FSP1 DAPI
30 μm
CKD+Reparixin
TEK-EGFP FSP1 DAPI

a

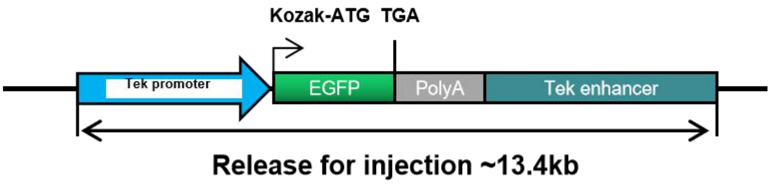

Kozak-ATG TGA
Tek promoter
EGFP
PolyA
Tek enhancer
Release for injection ~13.4kb

b

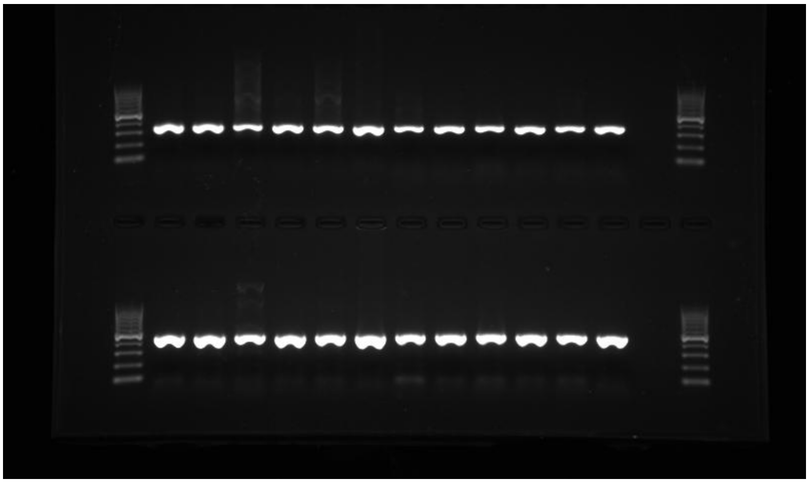

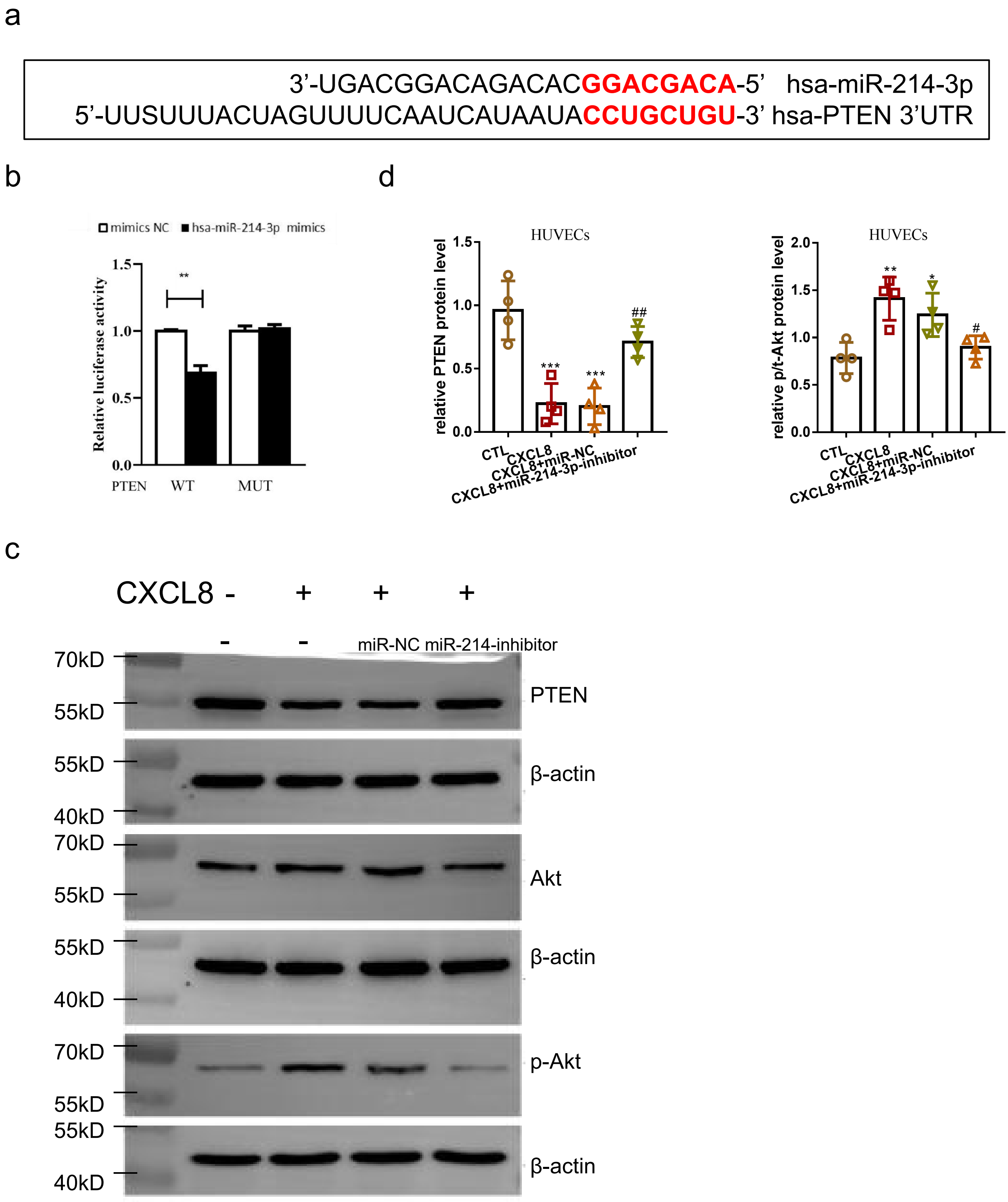
a
3’-UGACGGACAGACACGGACGACA-5’ hsa-miR-214-3p
5’-UUSUUUACUAGUUUUCAAUCAUAAUACCUGCUGU-3’ hsa-PTEN 3’UTR
b
mimics NC
hsa-miR-214-3p mimics
Relative luciferase activity
1.5
1.0
0.5
0.0
**
PTEN
WT
MUT
d
HUVECs
relative PTEN protein level
1.5
1.0
0.5
0.0
***
***
##
CTL
CXCL8
CXCL8+miR-NC
CXCL8+miR-214-3p-inhibitor
HUVECs
relative p/t-Akt protein level
2.0
1.5
1.0
0.5
0.0
**
*
#
CTL
CXCL8
CXCL8+miR-NC
CXCL8+miR-214-3p-inhibitor
c
CXCL8 - + + +
- - miR-NC miR-214-inhibitor
70kD
55kD
PTEN
55kD
40kD
β-actin
70kD
55kD
Akt
55kD
40kD
β-actin
70kD
55kD
p-Akt
55kD
40kD
β-actin

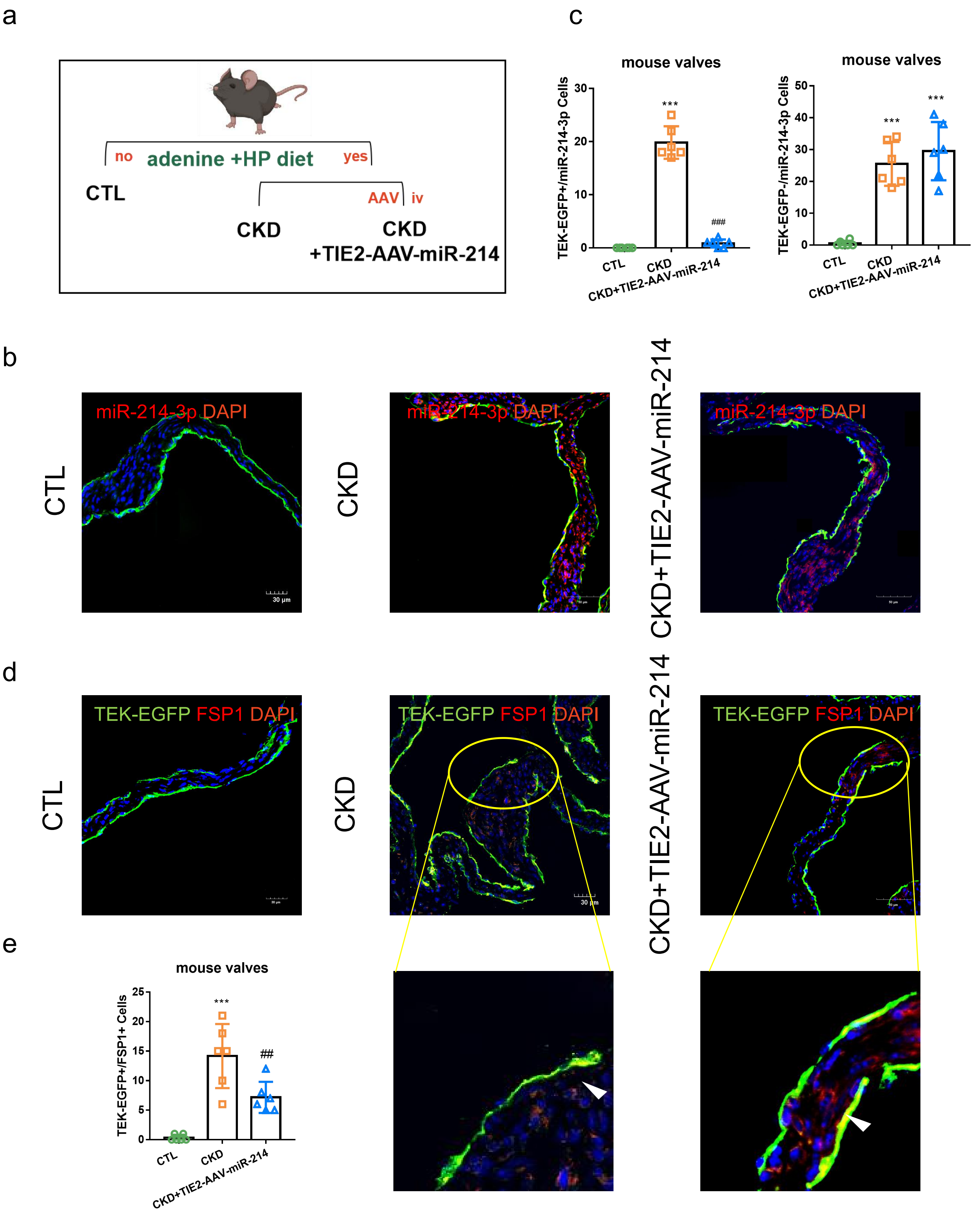

a
no
adenine +HP diet
yes
CTL
AAV
iv
CKD
CKD
+TIE2-AAV-miR-214
c
mouse valves
TEK-EGFP+/miR-214-3p Cells
***
###
CTL
CKD
CKD+TIE2-AAV-miR-214
mouse valves
TEK-EGFP-/miR-214-3p Cells
***
***
CTL
CKD
CKD+TIE2-AAV-miR-214
b
miR-214-3p DAPI
CTL
CKD
CKD+TIE2-AAV-miR-214
30 μm
d
TEK-EGFP FSP1 DAPI
CTL
CKD
CKD+TIE2-AAV-miR-214
30 μm
e
mouse valves
TEK-EGFP+/FSP1+ Cells
***
##
CTL
CKD
CKD+TIE2-AAV-miR-214
