# Supplementary figures and images for: High phosphorus mediated the release of C‐X‐C motif chemokine ligand 8 in valvular interstitial cells‐induced endothelial‐to‐mesenchymal transition via miR‐214/phosphatase and tensin homolog to promote valvular calcification in chronic kidney disease
Source: Clin Transl Med. 2022 May 23;12(5):e733. doi: 10.1002/ctm2.733 (PMC9126498; doi:10.1002/ctm2.733)

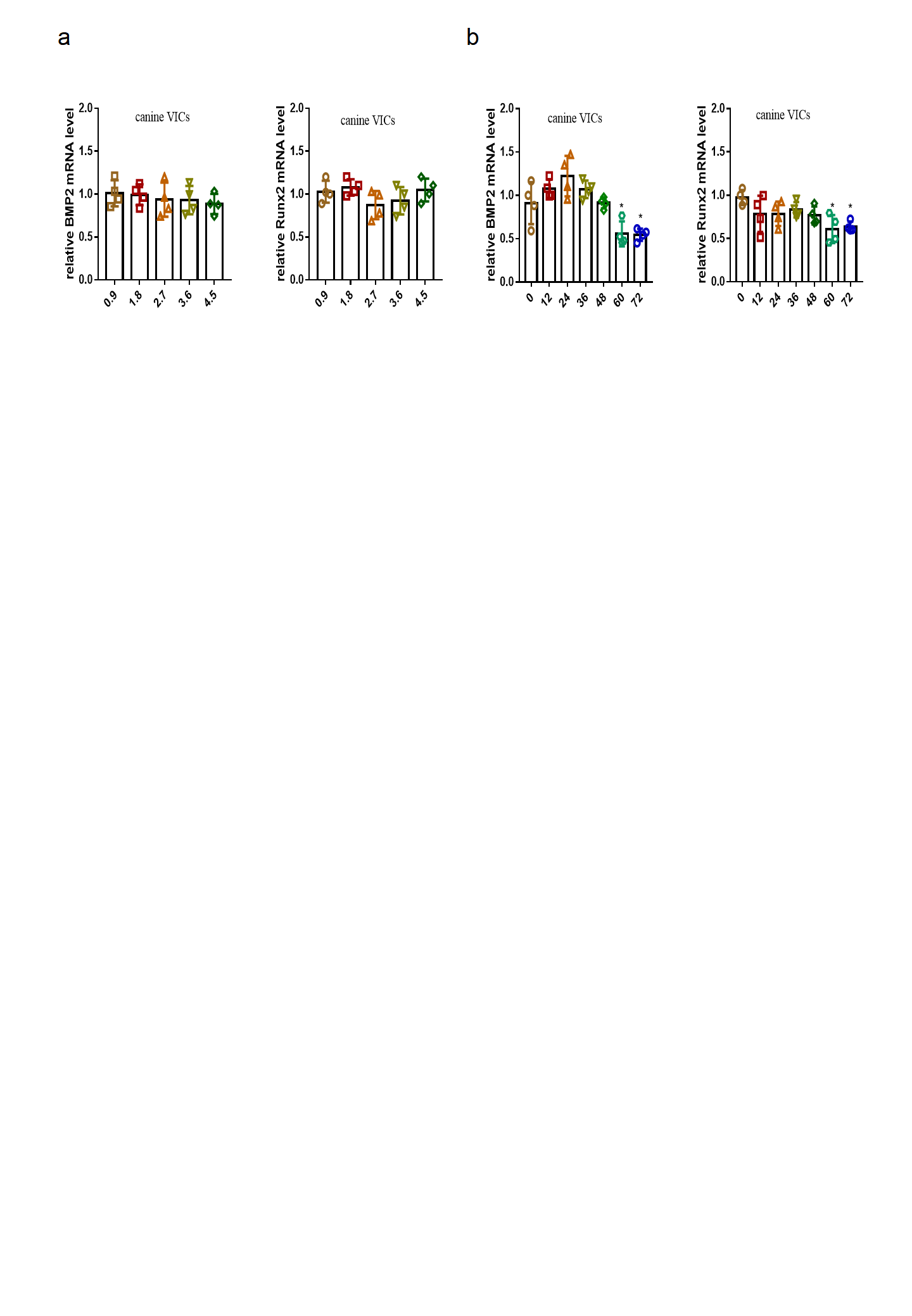

Supplement: Supplementary file 2 — SUPPORTING INFORMATION [file CTM2-12-e733-s007.png]

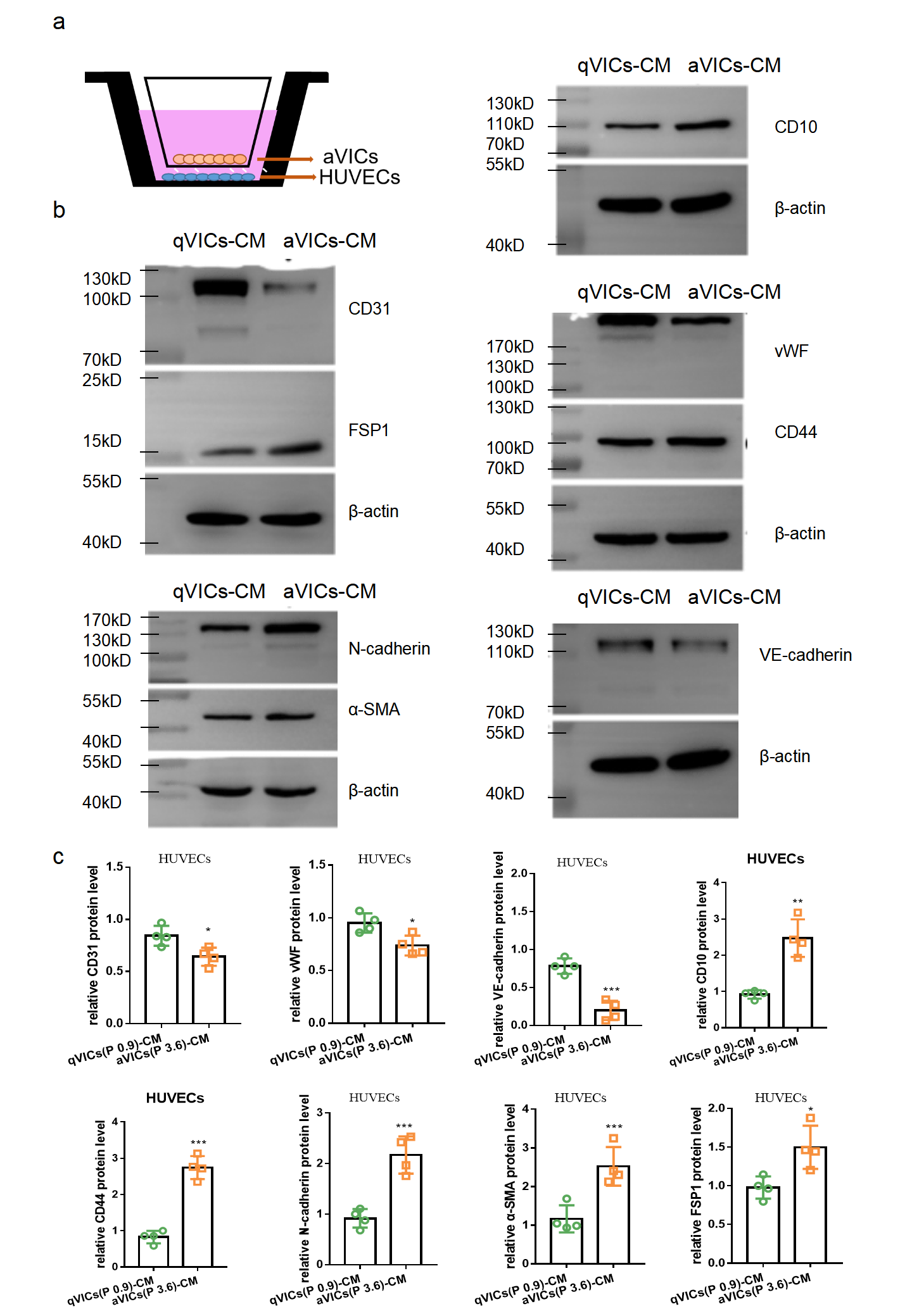

Supplement: Supplementary file 3 — SUPPORTING INFORMATION [file CTM2-12-e733-s006.png]

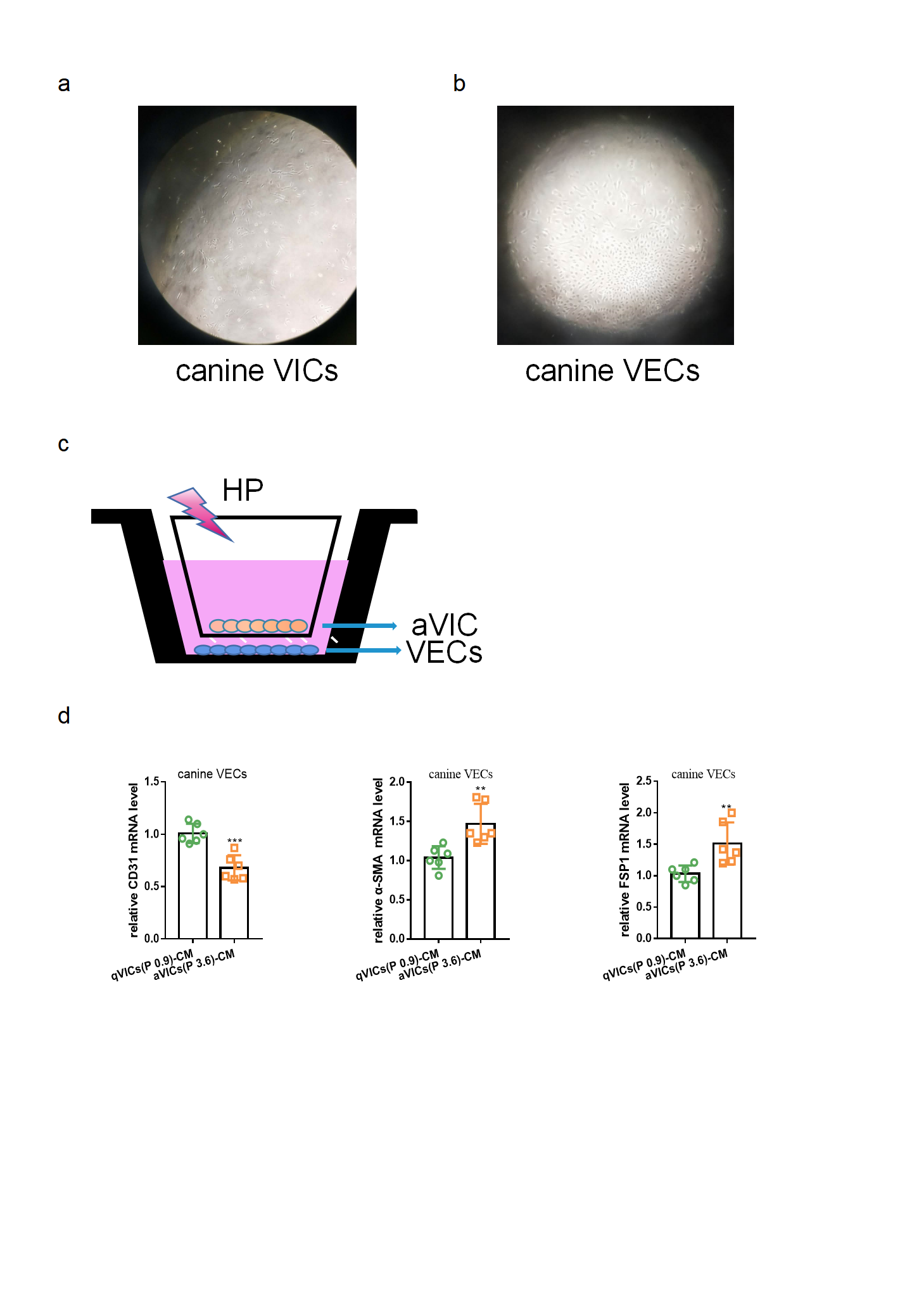

Supplement: Supplementary file 4 — SUPPORTING INFORMATION [file CTM2-12-e733-s010.png]

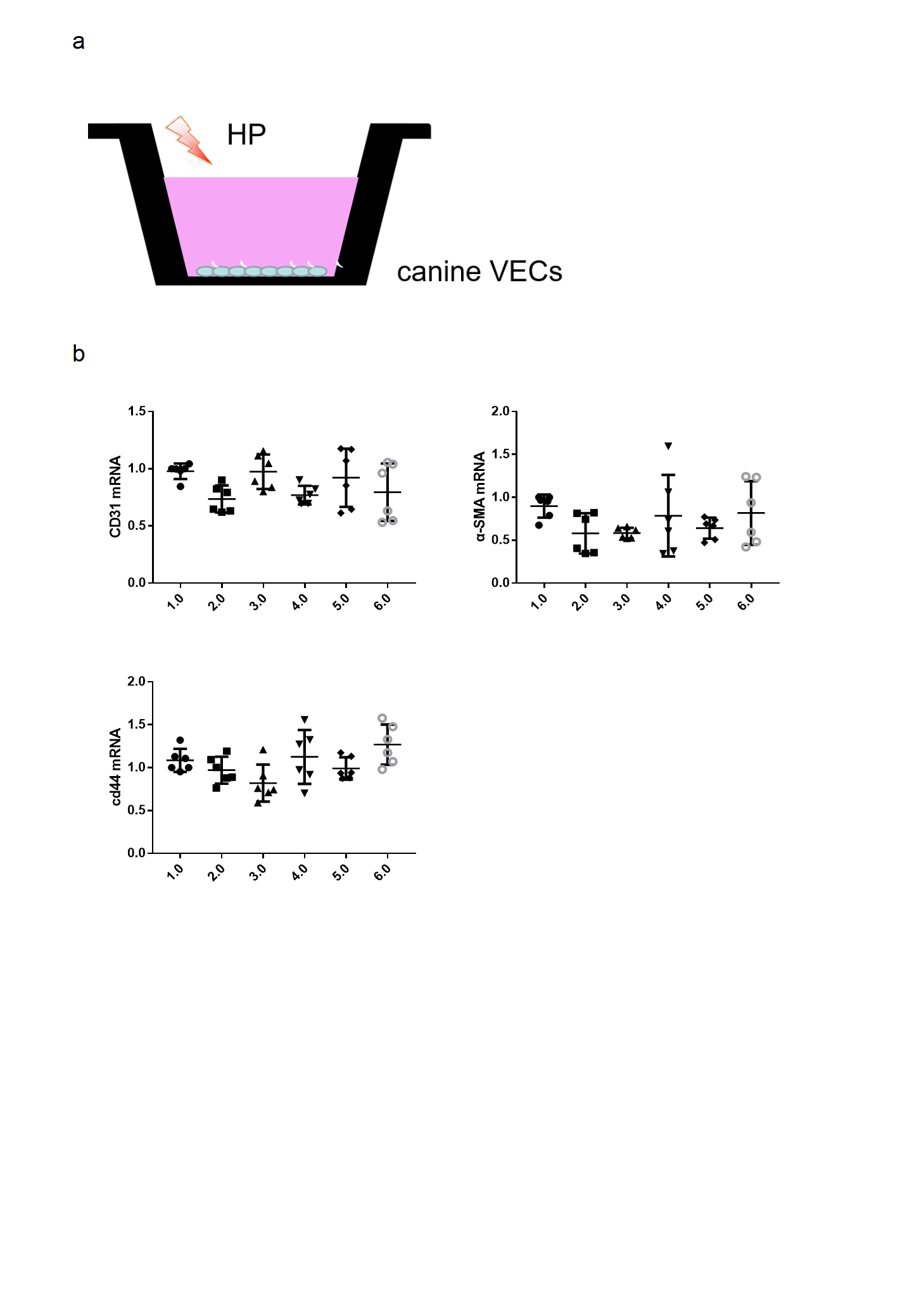

Supplement: Supplementary file 5 — SUPPORTING INFORMATION [file CTM2-12-e733-s013.png]

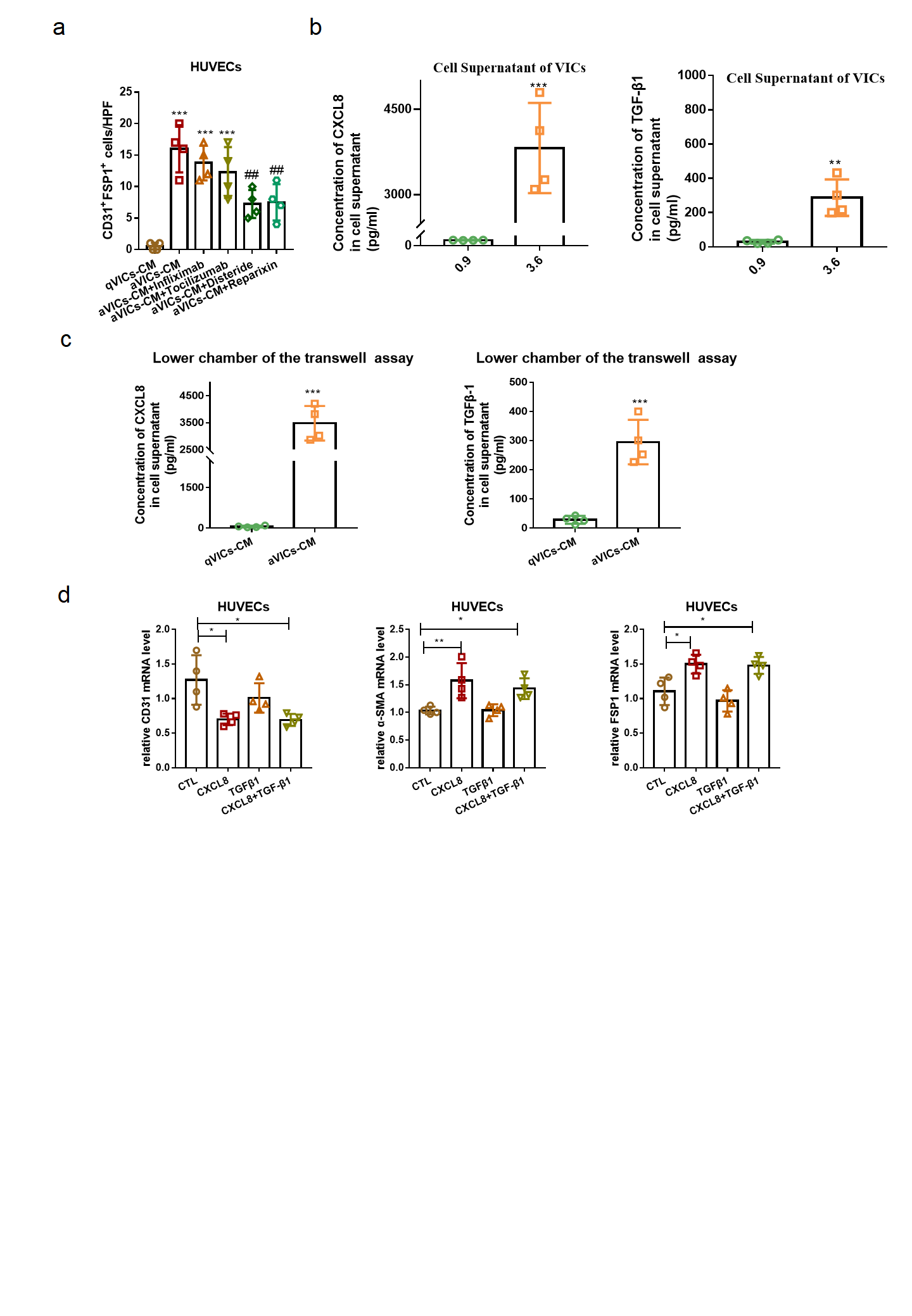

Supplement: Supplementary file 6 — SUPPORTING INFORMATION [file CTM2-12-e733-s005.png]

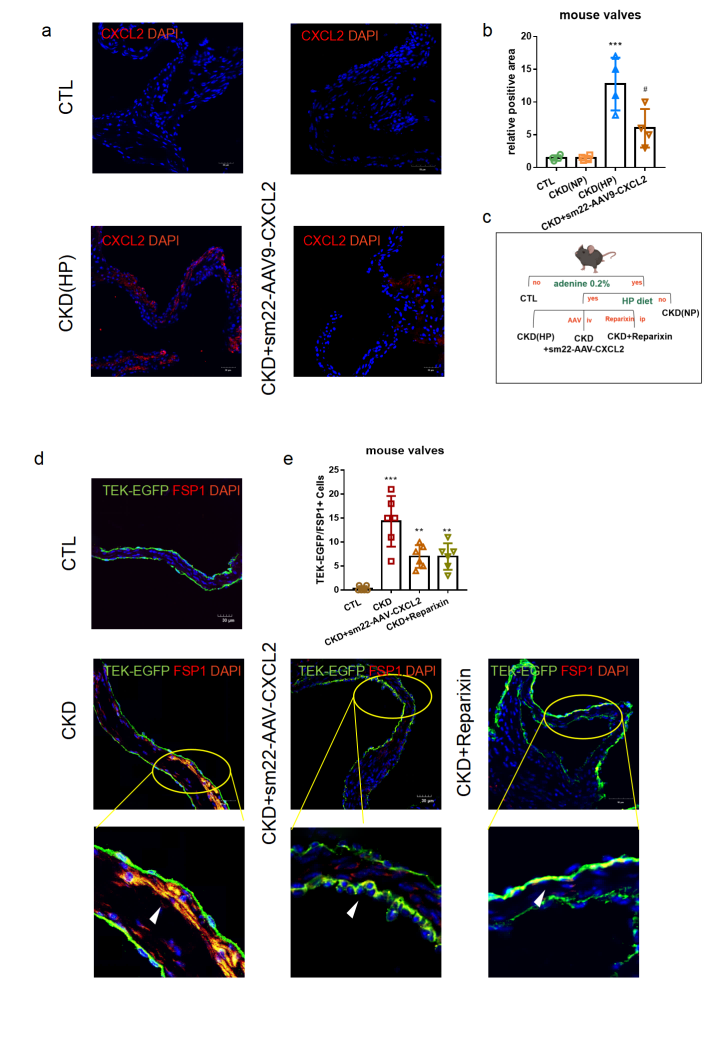

Supplement: Supplementary file 7 — SUPPORTING INFORMATION [file CTM2-12-e733-s004.png]

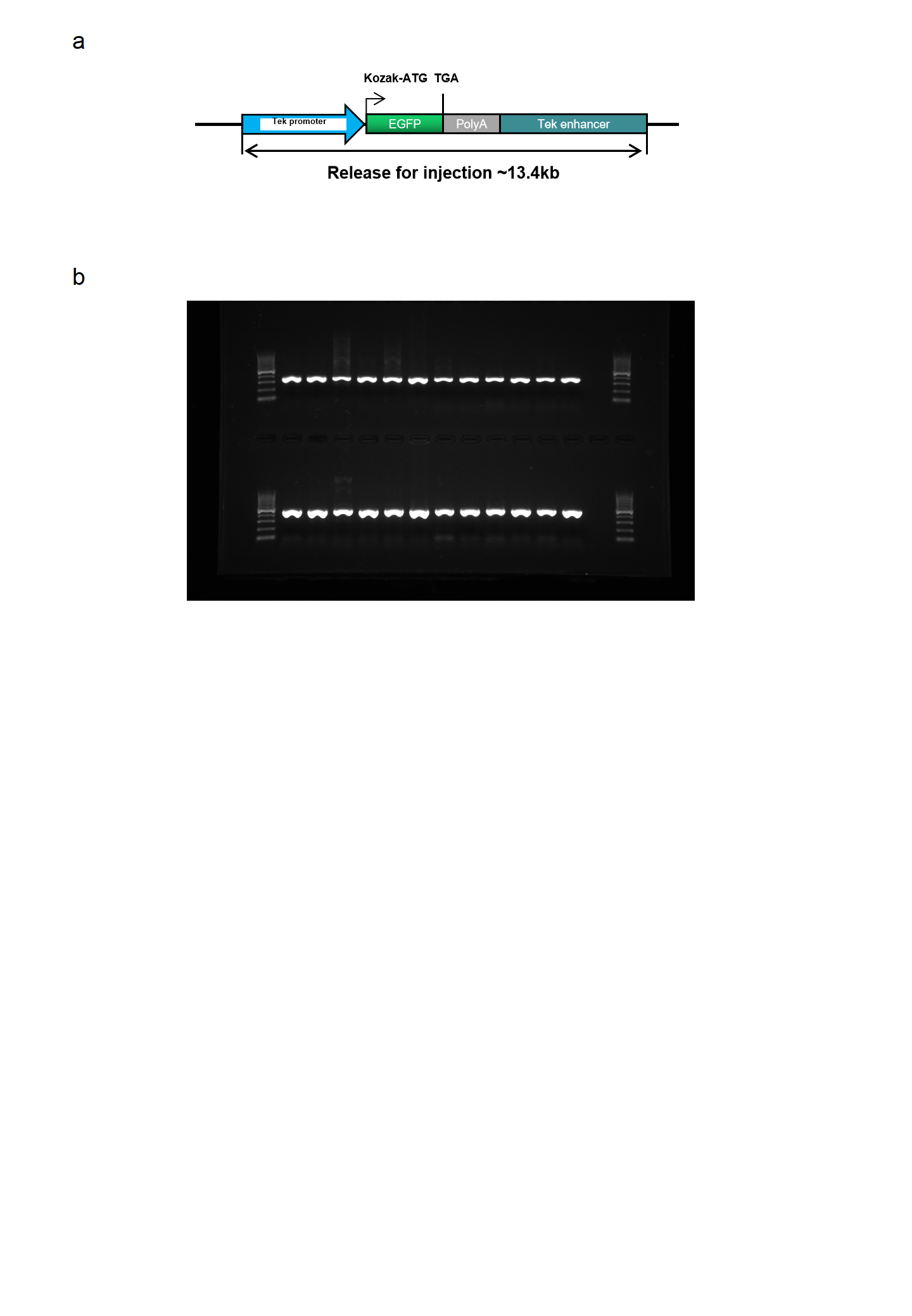

Supplement: Supplementary file 8 — SUPPORTING INFORMATION [file CTM2-12-e733-s003.png]

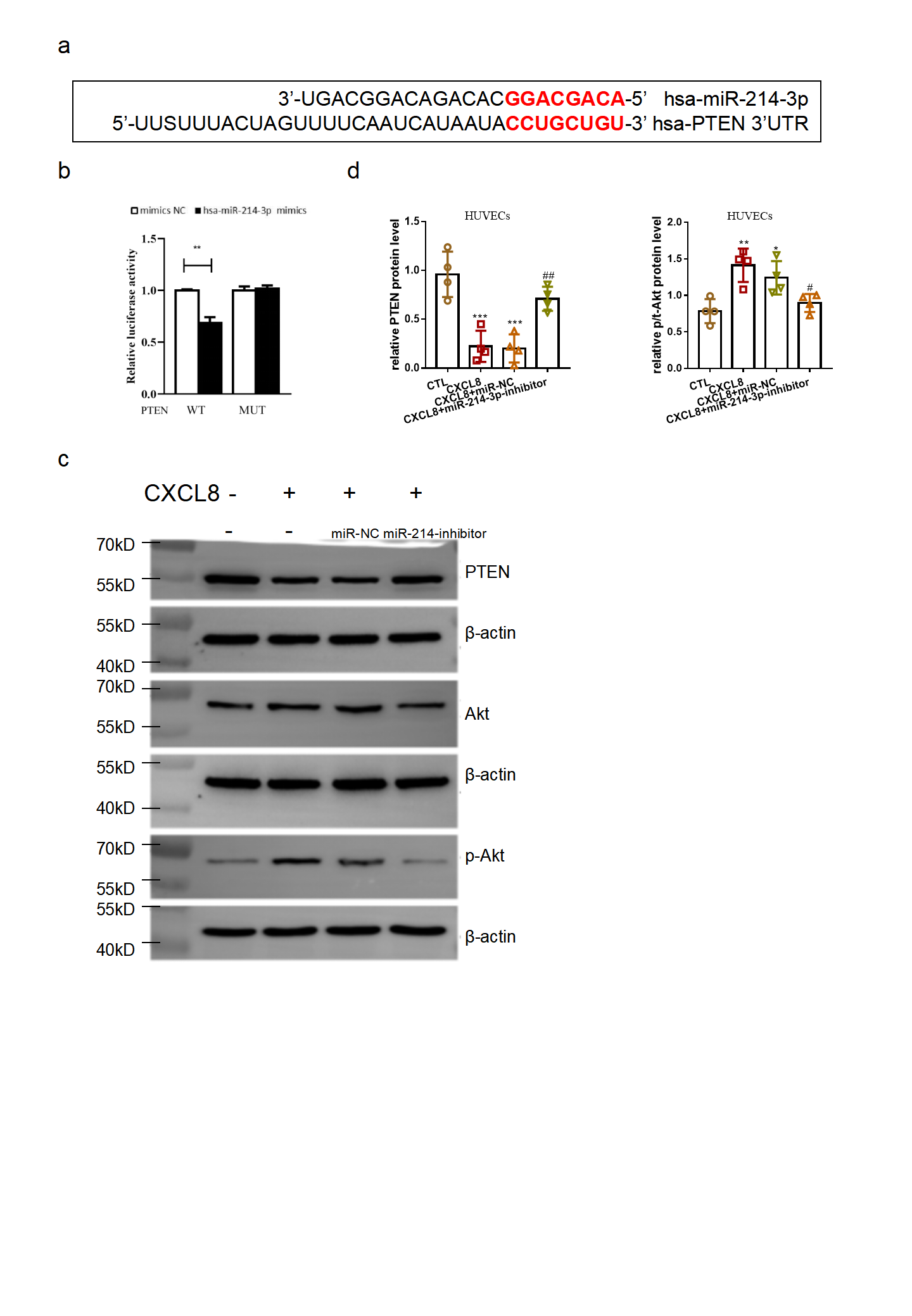

Supplement: Supplementary file 9 — SUPPORTING INFORMATION [file CTM2-12-e733-s001.png]

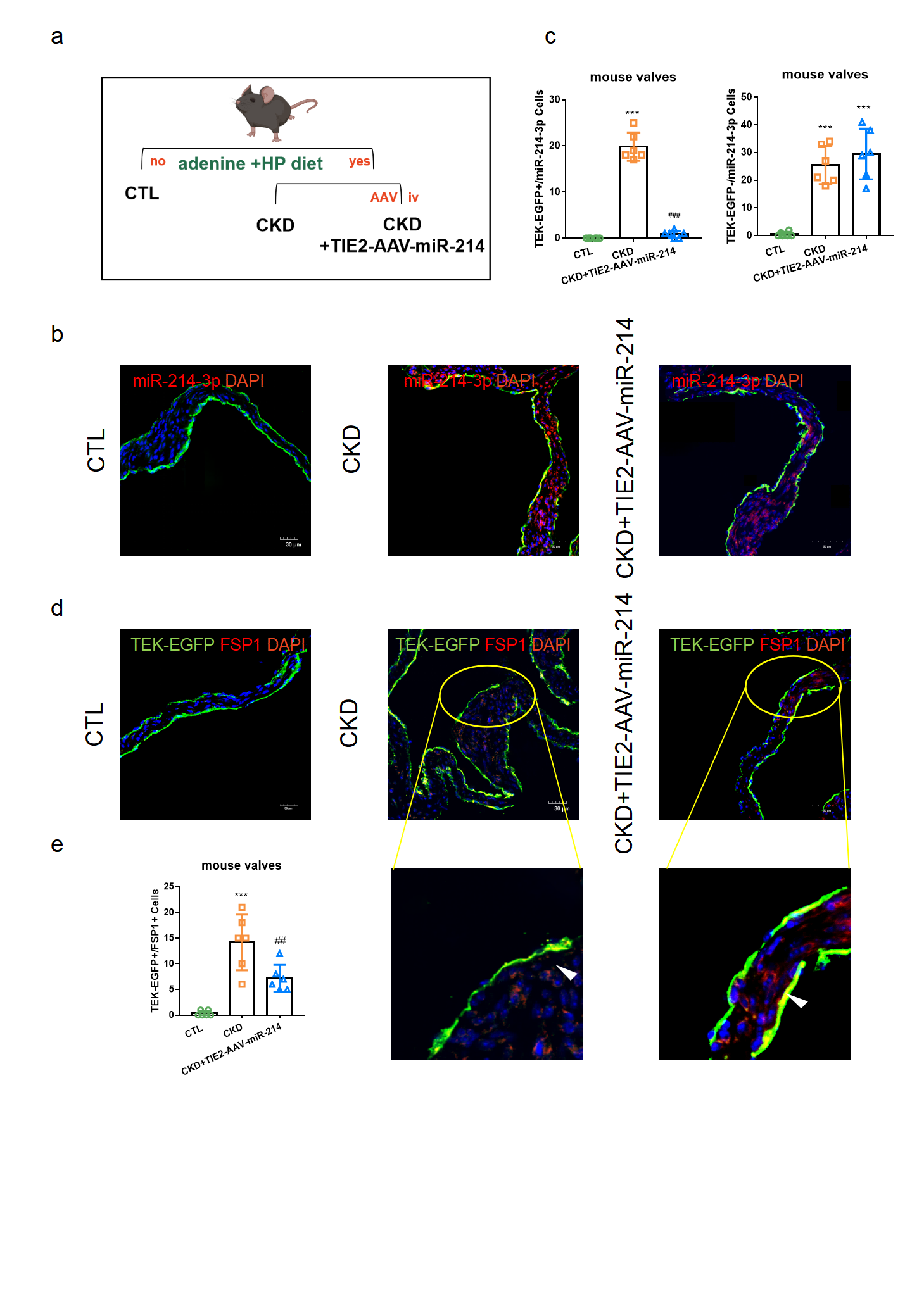

Supplement: Supplementary file 10 — SUPPORTING INFORMATION [file CTM2-12-e733-s002.png]
