# Supplementary material for: High phosphorus mediated the release of C‐X‐C motif chemokine ligand 8 in valvular interstitial cells‐induced endothelial‐to‐mesenchymal transition via miR‐214/phosphatase and tensin homolog to promote valvular calcification in chronic kidney disease
Source: Clin Transl Med. 2022 May 23;12(5):e733. doi: 10.1002/ctm2.733 (PMC9126498; doi:10.1002/ctm2.733)
Supplement: Supplementary file 11 — SUPPORTING INFORMATION [file CTM2-12-e733-s008.docx]

S1. HP could not induce aVICs transferred into oVICs.

a. HP (0.9, 1.8, 2.7, 3.6, 4.5mmol/L) was intervened in VICs for 24h. Each group was illustrated as means±SE and repeated 4 times. b. HP (3.6mmol/L) was intervened in VIC for 0h, 12h, 24h, 36h, 48h, 60h, and 72h. Each group was presented as means± SE and repeated 4 times.

S2. aVICs induced ECs EndMT.

HUVECs and canine VICs were used in this study. HUVECs were cocultured in a transwell assay in the presence of qVICs or aVICs (which were intervened by HP for 24hours before) for 48 hours. a. This is a schematic diagram with VICs in the upper chamber and HUVECs in the lower chamber. b-c. Endothelial markers and interstitial markers were examined in HUVECs. Each group was exhibited as means±SE and repeated 4 times. ^*^, *p*<0.05 *vs.* qVICs (P 0.9)-CM; ^**^, *p*<0.01 *vs.* qVICs (P 0.9)-CM; ^***^, *p*<0.001 *vs.* qVICs (P 0.9)-CM.

S3. aVICs induced VECs EndMT.

Canine VECs and canine VICs were used in this study. VECs were cocultured in a transwell assay in the presence of qVICs or aVICs (which were intervened by HP for 24hours before) for 48 hours. a. Cultured canine VICs. b. Cultured canine VECs. c. This is a schematic diagram with VICs in the upper chamber and VECs in the lower chamber. d. Endothelial markers and interstitial markers were examined in HUVECs. Each group was exhibited as means±SE and repeated 6 times. ^**^, *p*<0.01 *vs.* qVICs (P 0.9)-CM; ^***^, *p*<0.001 *vs.* qVICs (P 0.9)-CM.

S4. HP could not induce EndMT of VECs.

Canine VECs and canine VICs were used in this study. Canine VECs were cocultured in a transwell assay in the presence of qVICs or aVICs (which were intervened by HP for 24hours before) for 48 hours. a. This is a schematic diagram with VICs in the upper chamber and VECs in the lower chamber. b-c. Endothelial markers and interstitial markers were examined in VECs Each group was exhibited as means±SE and repeated 6 times.

S5. CXCL8 rather than TGFβ-1 released by aVICs induced ECs EndMT.

a. VICs were intervened by HP (0.9mmol/L and 3.6mmol/L) in a six-well plate, and the concentrations of CXCL8 and TGFβ1 in the cell supernatant were detected. Each group was presented as means±SE and repeated 4 times. ^***^, *p*<0.001 *vs.* 0.9. b. In the transwell assay, the concentrations of CXCL8 and TGFβ1 in the lower chamber were detected. Each group was illustrated as means±SE and repeated 4 times (t-test). ^***^, *p*<0.001 *vs.* aVICs-CM. d. According to a and b, CXCL8 (4ng/ml), TGF-β1 (300 pg/ml), and CXCL8+TGF-β1 were treated with canine VECs. Endothelial markers and interstitial markers were examined in canine VECs. Each group was displayed as means±SE and repeated 4 times. ^*^, *p*<0.05 *vs.* CTL; ^**^, *p*<0.01 *vs.* CTL.

S6. Inhibiting CXCL2 *(CXCL8 functional homolog in mouse)* expression /blocking CXCR1/CXCR2 (CXCL8 receptor) could attenuate valvular EndMT in CKD mouse.

The group of CTL and CKD (NP) yielded congruent data. Thus, data from the CTL group were shown. In the following, 'CKD' means 'CKD (HP)' if there is no special reference to 'CKD'. a-b. The data indicated the expression of CXCL2 in valves. The areas of red staining were counted. Each group was presented as means±SE and repeated 4 times. ^*^, *p*<0.05 *vs.* CTL; ^#^, *p*<0.05 *vs.* CKD (HP). c. The diagram of TEK^+^ mouse. d-e. The valvular endothelial cells expressing FSP1 were counted. Each group was exhibited as means±SE and repeated 6 times. ^**^, *p*<0.01 *vs.* CTL; ^***^, *p*<0.001 *vs.* CKD.

S7.

a. The construction strategy of Tek-EGFP-PolyA mouse; b. Identification of 1. Tek-EGFP-PolyA mouse.

S8. CXCL8 induced EndMT of ECa via miR-214-3p/PTEN/Akt pathway.

a. The site prediction of miR-214-3p with PTEN. b. The dual-luciferase assay miR-214-3p with PTEN. Each group was presented as means±SE and repeated 10 times (t-test). ^**^*p* <0 .01 *vs.* mimics-NC. c-d. The expressions of PTEN, pAKT, and tAKT in HUVECs were detected upon CXCL8 with/without miR-214-3p-inhibitor and OE-PTEN. Each group was exhibited as means±SE and repeated 4 times. ^**^, *p*<0.01 *vs.* CTL; ^***^, *p*<0.001 *vs.* CTL; ^#^, *p*<0.05 *vs.* CXCL8; ^##^, *p*<0.01 *vs.* CXCL8.

S9. Inhibiting miR-214-3p expression in VECs could attenuate valvular EndMT in CKD mice.

a. The diagram of TEK^+^ mouse. b-c. The VECs expressing miR-214-3p were counted to verify that TIE2-AAV-miR-214 could knock down miR-214 of VECs. Each group was exhibited as means±SE and repeated 6 times. ^***^, *p*<0.001 *vs.* CTL; ^###^, *p*<0.001 *vs.* CKD. d-e. The valvular endothelial cells expressing FSP1 were counted. Each group was presented as means±SE and repeated 6 times. ^***^, *p*<0.01 *vs.* CTL; ^##^, *p*<0.01 *vs.* CKD.
