# Supplementary material for: High phosphorus mediated the release of C‐X‐C motif chemokine ligand 8 in valvular interstitial cells‐induced endothelial‐to‐mesenchymal transition via miR‐214/phosphatase and tensin homolog to promote valvular calcification in chronic kidney disease
Source: Clin Transl Med. 2022 May 23;12(5):e733. doi: 10.1002/ctm2.733 (PMC9126498; doi:10.1002/ctm2.733)
Supplement: Supplementary file 12 — SUPPORTING INFORMATION [file CTM2-12-e733-s012.docx]

**Methods and Materials:**

1. **Cells**

**Canine aortic valvular interstitial cells (VICs):** Cells were isolated from 6 healthy canine aortic valves. Beagles were male aged 2-5 years. Local approval was granted by the institutional ethics review board. Beagles were anaesthetized by rapid intravenous injection of pentobarbital (100 mg/kg). The trimmed aortic valve was washed 5 times in PBS and then cut into small pieces. Then, the surface of the valves was wiped using sterile cotton to remove endothelial cells. Next, the valve was digested in collagenase I (20 ng/ml, sharp) solution for 30 minutes and placed in a 37℃, 5% CO_2_ environment in ECM (ScienCell, catalog number: 1001). VICs of passage3-5 were adopted in this study.

**Canine aortic valvular endothelial cells (VECs):** The separation process of aortic valves was the same as above. The specific steps were referred to previous paper^[1]^. The separated VECs were directly planted on the 6-well plate. VECs of passage 1 were adopted in this study.

**Umbilical vein endothelial cells (HUVECs):** Primary HUVECs were purchased from Sciencell and cultured in ECM. HUVECs of passages 3-5 were employed in this experiment.

1. **Transwell**

The co-culture system was established to investigate the cell dialogue between VICs and VECs. In the transwell cabin, VICs (4×10^^^4) were cultured in the upper chamber with a membrane pore size of 0.4μm for 24 hours with HP. Next, VICs in the upper chamber and HUVECs/VECs in the lower chamber were co-cultured. VICs secrete inflammatory mediators, which affect HUVECs/VECs. HUVECs/VECs in the lower chamber were collected after 48 hours of co-cultivation.

1. **Tek-EGFP-PolyA mouse**

Name of target gene (NCBI number): Tek (21687). The design strategy diagram is provided in S7.a. Identification sequence: ① 5’connector: F1: CCTTGGATGAAGGGCAAGATG, R1: AACTTGTGGCCGTTTACGTCG; ② 3’connector: F2: CCTGCTGTCCATTCCTTATTCCATA, R2: AGGAGGCAGCATCTGTCTACAAG. The results of mouse identification were presented in S7.b.

1. **CKD model**

The CKD model was constructed with an eight-week-old TEK mouse after the F3 generation and C57/Bj mouse. Specifically, a 0.2% adenine diet was provided for 6 weeks, followed by a high phosphorus diet (P, 1.8%)/normal phosphorus diet (P, 0.9%) for 10 weeks. Then, the CKD model can be established. The control group was given a normal-phosphorus diet for 16 weeks. After 12 weeks, the mice were euthanized by intraperitoneal injection of pentobarbital sodium 150 mg/kg. Finally, the blood supernatant was collected.

1. **Statistical analysis**

Data were expressed as mean ± standard deviation (SD) for each group. A two-tailed unpaired Student's t-test was performed to compare the two groups. One-way ANOVA was conducted to compare data with more than two groups. Afterward, the Bonferroni correction was completed for multiple comparisons. All analyses were conducted using GraphPad Prism 5.0. P<0.05 indicated statistical significance.

Reference：

1. Liu MM, Flanagan TC, Lu CC, French AT, Argyle DJ, Corcoran BM. Culture and characterisation of canine mitral valve interstitial and endothelial cells. Vet J. 2015; 204:32-39.
