# Supplementary material for: High phosphorus mediated the release of C‐X‐C motif chemokine ligand 8 in valvular interstitial cells‐induced endothelial‐to‐mesenchymal transition via miR‐214/phosphatase and tensin homolog to promote valvular calcification in chronic kidney disease
Source: Clin Transl Med. 2022 May 23;12(5):e733. doi: 10.1002/ctm2.733 (PMC9126498; doi:10.1002/ctm2.733)
Supplement: Supplementary file 13 — SUPPORTING INFORMATION [file CTM2-12-e733-s011.docx]

| Species | Gene | Forward primer (5'->3') | Reverse primer (5'->3') |
| --- | --- | --- | --- |
| Canine | TNF-α | TGCCTCAGCCTCTTCTCCTT | TTCGGGGTTTGCTACAACAT |
| Canine | IL-6 | TGTGAAGACAGCAAAGAGGC | ATTGAACCCAGATTGGAAGC |
| Canine | IL-8 | GAACTTCGATGCCAGTGTATAA | CGGATCTTGTTTCTCAGCCT |
| Canine | Tgf-β1 | AGAATGGCTGTCCTTTGATGT | GAACCCGTTAATGTCTACTTGC |
| Canine | GADPH | TGATGCTGGTGCTGAGTATGT | GGATGACTTTGGCTAGAGGAG |
| Canine | IL-1β | CACCAGTGAAATGATGGCTTAC | TGACACGAAATGCCTCAGACT |
| Canine | BMP2 | TGGAAGAACTGCCAGAAATG | ATAAACTCGTCAGTGGGGATAG |
| Canine | Runx2 | CAGACCAGCAGCACTCCAT | CAGCGTCAACACCATCATTC |
| Canine | α-SMA | GCCCTGGACTTTGAGAACGA | GATGAAGGACGGCTGGAACA |
| Canine | Smoothelin | CTCTAATGATGGCACGAGGAC | GATGACGACGAGGAAAAGGT |
| Canine | Vimetin | CTTGAGCGGAAAGTGGAATC | TGAGGTCAGGCTTGGAAACA |
| Canine | GADPH | TGATGCTGGTGCTGAGTATGT | GGATGACTTTGGCTAGAGGAG |
| Canine | CD31 | AAAAGCAGTCCCCAAAGCAA | CTTGGCCTTGGCTTTCTTCA |
| Canine | FSP1 | GGCTCTGGATGTGATGGTGTCT | TTTTGGAAGGCGGCTTCAT |
